# *In Vitro* and *In Vivo* Antifungal Efficacy and Safety of the *Ca*Def2.1_G27‑K44_ Peptide against the Neglected and Drug-Resistant Pathogen *Candida krusei*

**DOI:** 10.1021/acsbiomedchemau.5c00020

**Published:** 2025-05-13

**Authors:** Thomas Z. A. Guimarães, Érica O. Mello, Douglas R. Lucas, Filipe Z. Damica, Fadi S. S. Magalhães, Luís G. M. Basso, André O. Carvalho, Valdirene M. Gomes, Gabriel B. Taveira

**Affiliations:** 1 Laboratório de Fisiologia e Bioquímica de Microrganismos, Centro de Biociências e Biotecnologia, 218479 Universidade Estadual do Norte Fluminense Darcy Ribeiro, Campos dos Goytacazes, Rio de Janeiro 28013-602, Brazil; 2 Laboratório de Ciências Físicas, Centro de Ciência e Tecnologia, 218479 Universidade Estadual do Norte Fluminense Darcy Ribeiro, Campos dos Goytacazes, Rio de Janeiro 28013-602, Brazil

**Keywords:** antimicrobial peptide, fungal infections, *Galleria mellonella*, non-*albicans Candida* species, *Pichia kudriavzevii*, therapeutic potential

## Abstract

The growing threat of fungal infections, driven by increasing drug resistance, has become a major global health concern. Candidiasis, a common human infection, is associated with high mortality, particularly in invasive cases. Among non-*albicans Candida* (NAC) species, *Candida krusei* (renamed *Pichia kudriavzevii*) is of clinical importance because of its intrinsic resistance to fluconazole, complicating treatment options. This study evaluated the antifungal efficacy and safety of the bioinspired peptide *Ca*Def2.1_G27‑K44_ (CDF-GK) against NAC species, with a specific focus on *C. krusei*, through a series of *in vitro* and *in vivo* tests. CDF-GK effectively inhibited the growth of several yeast species, including *C. glabrata*, *C. guilliermondii*, *C. bracarensis*, and *C. nivariensis*, with MIC values ranging from 3.12 to 200 μM. The peptide demonstrated particularly strong activity against *C. krusei*, with an MIC_100_ of 25 μM, an MFC_100_ of 50 μM, and an IC_50_ of 5 μM, surpassing the effectiveness of fluconazole. Additionally, CDF-GK inhibited biofilm formation, caused 100% cell death within 1 h, permeabilized the cell membrane, interacted with ergosterol, induced oxidative stress, mitochondrial dysfunction, and vacuolar fragmentation, and entered the intracellular space of *C. krusei*. *In vivo* assays using *Galleria mellonella* larvae confirmed the low toxicity of CDF-GK, even at high concentrations, and significantly improved the survival of infected larvae with minimal activation of cellular and humoral immune responses. These findings indicate that CDF-GK holds great promise as a therapeutic agent for *C. krusei* infections, as it combines potent antifungal action with safety in both *in vitro* and *in vivo* models.

## Graphical abstract

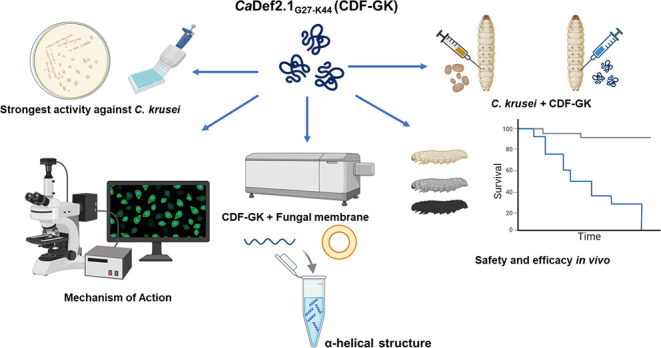

## Introduction

Fungal infections have long been underestimated by public health authorities, but recent data underscore their severe global impact, causing over 3.8 million deaths and affecting more than 1 billion people worldwide. These infections have a probable crude mortality rate of approximately 68%.^1,2^ The increasing prevalence of fungal infections is exacerbated by the continuous selective pressure from antifungal use, the presence of environmental residues of these agents, and their indiscriminate application in medical practice, which collectively contribute to the emergence of multiresistant strains. This issue is further complicated by the limited availability of clinical antifungal classes.^1,3^

One of the most significant infections is candidemia, a serious condition caused by yeasts of the genus *Candida*. Globally, the annual prevalence of candidaemia ranges from 250,000 to 700,000 cases, with mortality rates varying from 35% to 85%, depending on the *Candida* species involved.^4,5^ Candidemia typically develops as a secondary infection in immunocompromised individuals, with elderly individuals and children being particularly susceptible.^4,6,7^ Despite advancements in diagnostic tools and the benefits of early antifungal therapy, nearly half of invasive *Candida* infections remain undiagnosed, likely leading to an underestimation of their true incidence. As a result, candidaemia ranks as the fourth leading cause of death associated with hospital sepsis.^5,8–10^

In Brazil, the situation is even more concerning. The Ministry of Health lacks routine epidemiological surveillance for systemic mycoses, resulting in insufficient data on the prevalence, incidence, and impact of systemic candidiasis nationwide. Public tertiary hospitals in Brazil report an incidence rate of 2.49 cases of candidaemia per 1,000 hospital admissions, a figure significantly higher by a factor of 2–15 than those reported in the United States and Europe.^11^

While *Candida albicans* remains the primary etiological agent of candidemia, there has been evidence of an epidemiological shift over the past decades, with an increasing prevalence of non-*albicans Candida* (NAC) species, such as *Candida glabrata*, *Candida tropicalis*, *Candida parapsilosis*, and *Candida krusei* (recently renamed *Pichia kudriavzevii*). Collectively, these species account for 55% to 65% of candidaemia cases.^12,13^ Although *C. krusei* is detected in only 1.5% to 8% of clinical isolates, it is noteworthy for its high crude mortality rate of 57.9%, which is particularly concerning despite its lower incidence.^14–16^

Various antifungal agents, including polyenes, azoles, echinocandins, nucleoside analogs, and allylamines, are used to treat *Candida* infections. Fluconazole, in particular, is widely used for empirical therapy against candidiasis. However, *C. krusei* is intrinsically resistant to fluconazole, with more than 95% of clinical and veterinary isolates being unresponsive. Additionally, *C. krusei* shows reduced susceptibility to other azoles and polyenes, necessitating the use of alternative antifungal agents for effective treatment.^15,17,18^

Previously, our research group designed the antimicrobial peptide *Ca*Def2.1_G27‑K44_ (dubbed CDF-GK) on the basis of its physicochemical properties and explored its antimicrobial activity and low toxicity to mammalian cells. Comprising 18 amino acid residues with a global charge of +6, CDF-GK can form an α-helix in the presence of anionic membranes. It is active against *Candida* species and *Mycobacterium tuberculosis* *in vitro* and exhibits low cytotoxicity toward mammalian cells.^19^ In this study, we further explored the antifungal and antibiofilm properties of CDF-GK *in vitro* and *in vivo* using *Galleria mellonella* larvae as an infection model and demonstrated the safety and efficacy of this peptide in treating candidiasis caused by *C. krusei*.

## Materials and Methods

### Microorganisms

The yeasts *C. bracarensis* (ATCC 10154), *C. glabrata* (ATCC 90030), *C. guillermondii* (ATCC 6260), *C. krusei* (ATCC 6258), and *C. nivariensis* (ATCC 9983) were maintained on Sabouraud agar (1% peptone, 2% glucose, and 1.7% agar) (Merck) and preserved in the LFBM at the CBB of UENF, Campos dos Goytacazes, Rio de Janeiro, Brazil.

### Handling and Incubation Conditions for *G. mellonella* Larvae

*G. mellonella* larval colonies are maintained at LFBM/CBB/UENF, employing the diet outlined by Jorjão et al.^20^ Larvae in the sixth instar, weighing between 0.25 and 0.3 g and exhibiting no discernible melanization, were separated into groups of 10 or 15 within Petri dishes to be used in the assays. Before infection, the larval proleg was cleaned with 70% ethanol. Using 10 μL insulin syringes (Uniqmed, needle 8 mm × 0.30 mm, 5/16 in. × 30G), yeast and peptide suspensions were injected into the larvae. The assessment of larval mortality was conducted through visual inspection, considering factors such as color changes (melanization) and the absence of movement upon contact with tweezers.

### Yeast Growth Inhibition Assay

Cells from the different yeast species *Candida bracarensis*, *Candida glabrata*, *Candida guillermondii*, *Candida krusei* and *Candida nivariensis* (1 × 10^4^ cells/mL) were incubated in Sabouraud broth containing different concentrations of the CDF-GK peptide (200 μM to 1.56 μM), with the final assay volume adjusted to 100 μL. The assay was carried out in 96-well cell culture plates (Nunc) at 30 °C for a period of 24 h. Optical densities were measured at 620 nm after 24 h. Untreated yeast cells were used as a positive growth control, and the culture medium was used as a negative growth control. The minimum inhibitory concentration (MIC_100_) was defined visually as the lowest peptide concentration (in μM) at which 100% inhibition of yeast growth was observed within 24 h. The 50% inhibitory concentration (IC_50_) was defined as the peptide concentration (in μM) that caused 50% inhibition of yeast growth and was estimated via nonlinear regression analysis. The entire procedure was carried out in triplicate according to the method of Taveira et al.^19^ Amphotericin B (AmB) (25 to 0.19 μM) and fluconazole (FLZ) (200 to 1.56 μM) were also tested against *C. krusei* cells. The assay results were determined statistically via one-way ANOVA, with mean differences of *p < 0.05, **p < 0.01, ***p < 0.001, and ****p < 0.0001 considered significant. Analyses were performed with Prism software (version 8.0.2).

### Yeast Cellular Viability Analysis

After the yeast growth inhibition assay, the entire contents of the wells containing the MIC_100_ or twice the MIC_100_ for all yeast tested were washed in Sabouraud broth and, via a Drigalski loop, evenly spread on a Petri dish containing Sabouraud agar. The plates were subsequently incubated at 30 °C for 24 h. Control cells were considered viable; that is, in the appropriate medium, they divided and formed colonies. The development of colonies indicates fungistatic action, while the absence of colony development indicates the fungicidal action of the peptide. The lowest concentration of peptide, in μM, with fungicidal action was considered MFC_100_, whose interpretation was based on the absence of colony growth after plating. This assay was based on the method described by Soares et al.^21^ The experiments were performed in triplicate.

### Kinetics of *C. krusei* Cell Death Induced by CDF-GK

After the viability of yeast treated with the CDF-GK peptide was analyzed, an assay was carried out to determine the minimum period necessary for the peptide, at the MFC_100_ concentration, to reduce *C. krusei* cell viability. This assay was carried out as described in the section “Yeast cellular viability analysis”, with the exception that a volume of 10 μL from each sample was transferred to Sabouraud agar plates at 0, 0.5, 1, 3, 6, 9, 12, 15, 18, 21, and 24 h intervals. Treated and untreated plates were incubated at 30 °C for 24 h. The colony-forming units (CFUs) were counted, and the percentage of cell survival was quantified by means of CFU/mL. The assay results were determined statistically via one-way ANOVA, with mean differences of ****p < 0.0001 considered significant. Analyses were performed with Prism software (version 8.0.2).

### Biofilm Formation Inhibition Assays

For the biofilm formation inhibition assays, a 200 μL aliquot of a suspension containing 2 × 10^7^ cells/mL *C. krusei* in BHI broth was added to each well of a 96-well microplate.^22^ The microplate was then incubated at 37 °C for 2 h to allow for cell adhesion. Following this period, each well was washed twice with sterile PBS to remove nonadherent cells. The peptide CDF-GK was diluted in BHI broth to obtain concentrations equivalent to the previously determined MIC, 2 × MIC, 4 × MIC, and 6 × MIC for planktonic cells. After different concentrations of CDF-GK were added to the wells, the plates were further incubated for 24 h at 37 °C. The same procedure was carried out for AmB, which was used as a positive control. Following the 24-h incubation with CDF-GK and AmB, the culture medium was removed, and the wells were washed twice with PBS to remove planktonic cells. The adhered biofilms were stained for 30 min with 200 μL of crystal violet at a final concentration of 0.1%. Excess stain was removed, and the biofilm was washed once with 200 μL of PBS. To release the retained crystal violet from the biofilm cells, 200 μL of 1% SDS in 50% ethanol was added, and the cellular material was resuspended by pipetting.^23^ The absorbance was measured at 490 nm via a microplate reader. The data presented represent the means of three independent experiments.

### Membrane Permeabilization

Membrane permeabilization in *C. krusei* was assessed through fluorescence microscopy using a SYTOX Green probe. The procedure followed the methodology outlined in the section “Yeast growth inhibition assay”, with the following adjustments: yeast cells were treated with 5 μM (IC_50_) CDF-GK for 24 h. Both the control (untreated cells), the positive control (Triton X-100, 0.1%), and peptide-treated cells were then incubated with a 0.2 μM solution of the SYTOX Green fluorescent probe for 10 min at 30 °C. Subsequent analysis was conducted via differential interference contrast (DIC) on an optical microscope (Axioplan A2, Zeiss) equipped with a fluorescence filter set for fluorescein detection (excitation wavelengths of 450–490 nm; emission wavelength of 500 nm).^24^ The positive control was used to optimize the excitation intensity and exposure time parameters during fluorescent image acquisition. These settings were then uniformly applied to all experimental treatments to ensure consistency.

### Small Unilamellar Vesicle (SUV) Preparation

The lipids 1-palmitoyl-2-oleoyl-*sn*-glycero-3-phosphocholine (POPC), 1-palmitoyl-2-oleoyl-*sn*-glycero-3-phosphoethanolamine (POPE), 1-palmitoyl-2-oleoyl-*sn*-glycero-3-phospho-L-serine (POPS), chicken egg sphingomyelin (SM), ergosterol (Erg), and cholesterol (Chol) were purchased from Avanti Polar Lipids, Inc.

SUVs were prepared as described elsewhere.^25^ Briefly, phospholipids from chloroform stock solutions were mixed at desired molar ratios in a glass tube and dried under a gentle stream of nitrogen gas to form a thin lipid film. The film was further dried under vacuum for at least 1 h to ensure complete removal of residual organic solvent. The film was hydrated with 5 mM HEPES buffer (pH 7.0) at room temperature, followed by six freeze–thaw cycles to promote vesicle formation and ensure homogeneous dispersion. The resulting suspension was sonicated on ice using a Sonics Vibra-Cell VCX 500 (Sonics & Materials, Inc.) tip sonicator for 1 min (5 s on and 10 s off) at 20% amplitude, facilitating the formation of SUVs. To remove any large insoluble aggregates and potential metallic particles released from the sonicator tip, the samples were centrifuged at 13,000 rpm for 10 min using a benchtop centrifuge (Eppendorf 5424R). Peptide stock solutions were prepared in ultrapure water and diluted into SUVs immediately prior to analysis to achieve the desired peptide-to-lipid molar ratio.

### Circular Dichroism (CD)

CD measurements were conducted at 30 and 37 °C on a Jasco J-815 spectrometer. Spectra were recorded in a 1 mm path-length quartz cuvette at 50 nm/s over the wavelength range of 350 to 190 nm. The data pitch and bandwidth were set to 1 and 2 nm, respectively. Peptide samples were prepared at a concentration of 30 μM in 5 mM HEPES buffer, pH 7.0. Spectra were acquired in the absence and presence of SUVs with varying lipid compositions. The following artificial model membranes were used to mimic distinct biological membranes: (i) POPC/SM/Chol (40/30/30 mol %) to simulate the mammalian plasma membrane; (ii) POPC/POPE/POPS (40/30/30 mol %) to represent a sterol-free fungal membrane; (iii) POPC/POPE/POPS/Erg (28/21/21/30 mol %) and POPC/POPS/Erg (40/30/30 mol %) to model the *Candida* genus membrane containing ergosterol; and (iv) POPC/POPE/POPS/Chol (28/21/21/30 mol %) to evaluate the impact of cholesterol replacing ergosterol in a fungal-like membrane. SUVs were prepared at a final lipid concentration of 450 μM, ensuring minimal light scattering and absorption flattening effects, with the photomultiplier tube voltage maintained below 600 V throughout the experiments. For each sample, 10 scans were accumulated and averaged. Data were processed using the CDToolX software.^26^ The average of the best scans for each measurement was subtracted from the blank (buffer solution or SUV), zeroed using the appropriate baseline region, and converted to mean residue ellipticity (MRE), θ, expressed in deg·cm^2^·dmol^–1^. Secondary structure content was estimated using DichroWeb,^27^ applying various algorithms and data sets. The normalized root mean squared deviation (NRMSD), along with visual inspection of the computed spectra, was used to assess the quality of the fit, ensuring accurate structural estimations.^28^

### Effects of CDF-GK on ROS Induction in *C. krusei*

To evaluate the ability of CDF-GK to induce oxidative stress, the fluorescent probe 2’,7’-dichlorofluorescein diacetate (H_2_DCFDA) was used to measure intracellular reactive oxygen species. The test was carried out as described in the section “Yeast growth inhibition assay”, with the following modifications: cells were incubated with 5 μM (IC_50_) CDF-GK for 24 h. A positive control was performed with 3% acetic acid. After 24 h of incubation, the control and treated cells were incubated with 20 μM H_2_DCFDA for 30 min at 30 °C and analyzed via DIC with an optical microscope (Axioplan A2, Zeiss) equipped with a fluorescence filter set for fluorescein detection (excitation wavelengths of 450–490 nm; emission wavelength of 500 nm).^29^

### Mitochondrial Membrane Potential (Δψ_m_)

To evaluate the Δψ_m_, we used 5,5′,6,6′-tetrachloro-1,1′,3,3′-tetraethylbenzimidazolocarbocyanine iodide (JC-1). The test was carried out as described in the item “Yeast growth inhibition assay”, with the following modifications: the cells were incubated with 5 μM (IC_50_) CDF-GK for 24 h. After this incubation period, the cell suspension was centrifuged at 2500 rpm for 15 min, washed once in 500 μL of PBS (10 mM NaH_2_PO_4_, 0.15 M NaCl), pH 7.4, and resuspended in 50 μL of PBS. After the cells were washed, 2 μM JC-1 dye was added, and the mixture was incubated for 30 min at 30 °C. The negative control cells (incubated without CDF-GK) were subjected to the same treatment as the cells treated with the peptide, and 1% Triton X-100 was used as the positive control. The cells were analyzed via DIC via an optical microscope equipped with a fluorescence filter to detect fluorescein (excitation wavelength: 450–490 nm; emission wavelengths: 530 and 590 nm).^30^

### Analysis of *C. krusei* Vacuolar Membranes

Vacuolar mapping of *C. krusei* was performed using the FM4-64 probe. FM4-64 is a lipophilic styryl dye that does not permeate cell membranes but instead intercalates into the plasma membrane and is then taken into cells by endocytosis, allowing labeling of vacuolar membranes. The test was carried out as described in the item “Yeast growth inhibition assay”, with the following modifications: *C. krusei* cells were incubated with 5 μM (IC_50_) CDF-GK for 24 h and then treated with FM4-64 for 1 h at 30 °C. The cells were subsequently washed and centrifuged with PBS twice to remove free FM4-64. The control was treated only with FM4-64.^31^ The cells were analyzed via DIC with an optical microscope (Axioplan A2, Zeiss) equipped with a fluorescence filter set with an absorption/emission wavelength of 585/590 nm.

### Time Lapse

Before conducting the time-lapse assay with the peptide conjugated to 5-FAM, its antifungal activity was validated through an MFC_100_ assay against *C. krusei*. This assay was carried out as described in item “Yeast cellular viability analysis”.

*C. krusei* cells were treated with CDF-GK coupled to 5-carboxyfluorescein (5-FAM) to investigate the interaction of CDF-GK with *C. krusei* cells and its ability to enter the intracellular space at a concentration of the fungicide (50 μM). Initially, *C. krusei* cells were cultivated in Sabouraud broth (Merck Millipore, Brazil) for 16 h at 30 °C. After the growth period, the cells were quantified in a Neubauer chamber (Laboroptik, United Kingdom) under an optical microscope (Axioplan A2, Carl Zeiss) to prepare an inoculum with 1 × 10^6^ cells/mL. For the time course experiments, aliquots of 200 μL of cell inoculum were transferred to 1.5 mL microcentrifuge tubes. Before the addition of CDF-GK coupled to 5-FAM (5-FAM-CDF-GK), the cells were pretreated with 10 μL of Calcofluor White (Calcofluor White M2R, 1 g/L; Evans Blue, 0.5 g/L; Sigma) for 10 min. After this step, the cells were transferred to microscopy slides, followed by the addition of 50 μM 5-FAM-CDF-GK. Immediately after the addition of 5-FAM-CDF-GK, the cells were monitored via a Zeiss LSM 710 Laser Scanning Confocal Microscope (Carl Zeiss, Germany) with a Plan Apochromat × 63/1.4 objective and scan intervals every 27 s. The images were created from the z-stack series of confocal planes with Zen Lite Edition 2011 (Zeiss). For the detection of the fluorescent probes, we used a set of fluorescence filters with excitation at 365 nm/emission at 397 nm for Calcofluor White and excitation at 450–490 nm/emission at 500 nm for the detection of 5-carboxyfluorescein.

### Effect of *In Vivo* Toxicity of CDF-GK on *G. mellonella*

The assay was performed as described by Mylonakis et al.,^32^ with modifications. Fifteen last-instar *G. mellonella* caterpillars with similar weights (between 250 and 300 mg) and sizes were used in each of the three treatment groups with CDF-GK. Insulin syringes were used to inject 10 μL of each concentration of CDF-GK (1000, 500, and 250 μM) into the hemocoel of each larva through the last proleg. Two groups were included as controls for the general viability of the larvae: one group was inoculated with PBS, and the other group sustained injury only from the injection needle. After injection, the larvae were incubated in Petri dishes at 37 °C, and the number of dead larvae was counted every 24 h for a period of 168 h. Larvae were considered dead when they showed no movement in response to touch. Survival percentage curves were plotted, and estimates of differences in survival (log-rank Mantel–Cox and Breslow–Wilcoxon tests) were analyzed via the Kaplan–Meier method via GraphPad (version 8.0.2).

### *In Vivo* Evaluation of the Therapeutic Activity of CDF-GK

To evaluate the effects of CDF-GK on *C. krusei* infection, the lethal concentration was initially determined by injecting serial dilutions of the fungal suspension into *G. mellonella* larvae. Yeast cells were centrifuged and washed with 0.9% NaCl. The cell density was standardized to 10^9^ cells/mL by spectrophotometry (590 nm), and concentrations of 10^3^ to 10^7^ cells/larva were used in the assay. A total of 10 μL of each standardized cell suspension was inoculated into the hemocoel of each larva through the last left proleg to determine the minimum lethal cell density. After inoculation, the larvae were incubated in Petri dishes at 37 °C, and the number of dead larvae was counted every 24 h for 168 h. Following the determination of the lethal cell concentration, larvae were inoculated with 10 μL of PBS containing 10^6^ *C. krusei* cells/larva via the last left proleg. After 30 min, CDF-GK (50 or 100 μM) or AmB (1.56 or 3.12 μM) was injected into the last right proleg to avoid cross-interference. PBS-injected larvae served as the controls. The larvae were incubated in Petri dishes at 37 °C, and the number of dead larvae was counted every 24 h for a period of 168 h. The larvae were considered dead when they did not show any movement to the touch at the end of 168 h.^33^ Every assay was performed in triplicate, and each independent experiment yielded similar results. The data presented here are from a representative experiment. Survival percentage curves were plotted, and estimates of differences in survival (log-rank Mantel–Cox and Breslow–Wilcoxon tests) were analyzed via the Kaplan–Meier method via GraphPad Software (version 8.0.2).

### Quantification of Hemocytes

The density of *G. mellonella* hemocytes was analyzed after 3, 6, and 24 h of inoculation, or lack thereof, with the yeast *C. krusei*. Three groups with five larvae each were used: 1-larvae only injected with PBS (PBS); 2-larvae inoculated with 10^6^ *C. krusei* cells/larva and treated with PBS (*C.k* + PBS); and 3-larvae inoculated with 10^6^ *C. krusei* cells/larva and treated with 100 μM CDF-GK (*C.k* + CDF-GK). Before hemolymph extraction, the larvae were cleaned with a 70% ethanol swab. Then, 10 μL of hemolymph from each larva in each group was collected separately after piercing the last proleg with an insulin needle, added to microcentrifuge tubes, diluted with insect physiological saline (IPS; 150 mM NaCl, 5 mM KCl, 100 mM Tris/HCl, 10 mM EDTA, 30 mM sodium citrate, pH 6.9) on ice at a 1:10, and centrifuged at 800 × *g* and 4 °C for 5 min. After centrifugation, the pellets were resuspended in ice-cold IPS, and hemocytes were quantified in a Neubauer chamber (Laboroptik).^33^

### Melanization Quantification

The quantification of melanin *in* *G. mellonella* after 3, 6, and 24 h of inoculation, or when not inoculated with the yeast *C. krusei* was carried out as described in the section “Quantification of hemocytes”, with the following modifications: after different incubation times, 10 μL of hemolymph from each larva in each group was collected separately by piercing the last proleg with an insulin needle and adding it to microcentrifuge tubes. The hemolymph was diluted with IPS buffer at a 1:10 ratio and centrifuged at 4500 g and 4 °C for 5 min. The supernatant of each sample was placed in a 96-well microdilution plate, and the optical density was determined with a spectrophotometer at 405 nm.^33^

## Results

### Antifungal Activity against Non*-Albicans* *Candida* Species

We tested the bioinspired peptide CDF-GK against non-*albicans Candida* (NAC) species at concentrations ranging from 200 to 1.56 μM. CDF-GK exhibited significant antifungal activity against all tested yeasts at a concentration of 3.12 μM, except for *C. glabrata* and *C. nivariensis*, where significant inhibition was observed only at concentrations of 6.25 and 25 μM, respectively (Figure 1). Among the NAC species, the strongest inhibitory activity was observed against *C. krusei* (renamed *Pichia kudriavzevii*), with the lowest MIC_100_ (25 μM) and IC_50_ (5 μM) values (Table 1).

**Figure 1:**
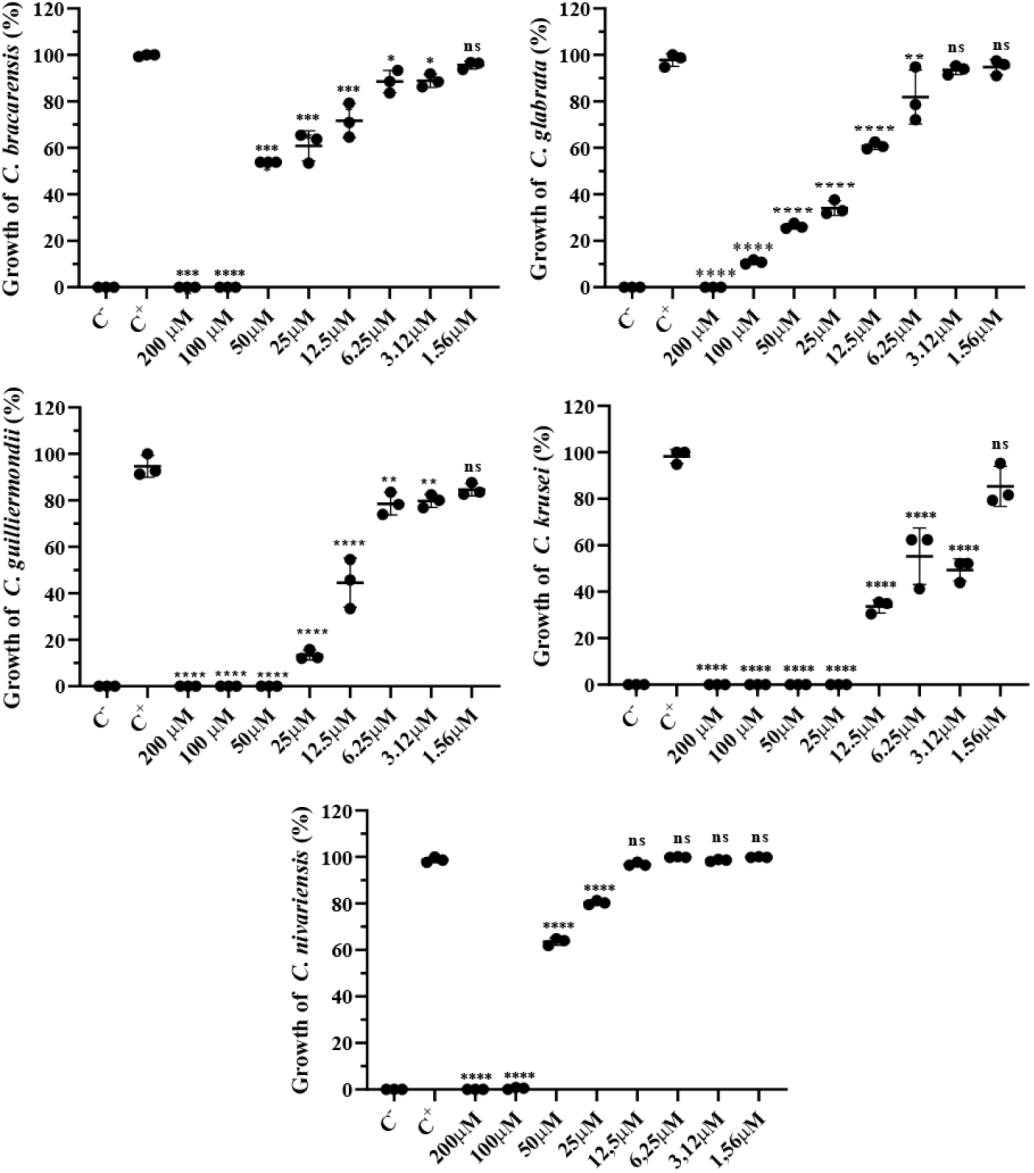
Anti-*Candida* activity of CDF-GK. Effect of CDF-GK on the growth of *C. glabrata*, *C. krusei*, *C. nivariensis*, *C. guilliermondii*, and *C. bracarensis* at concentrations ranging from 200 to 1.56 μM after 24 h of incubation. Data represent the mean ± SD (bars) with individual replicates (scatter points; *n* = 3). The assay is representative of an independent assay out of three. Asterisks indicate significant differences: *p < 0.05, **p < 0.01, ***p < 0.001, and ****p < 0.0001 between treatments and positive control (C^+^). (ns) indicates not significantly different.

**Table 1:** Antifungal Properties of CDF-GK against Non-*Albicans Candida* Species

MIC^a^/MFC^b^/IC_50_ ^c^ (μM)	CDF-GK	FLZ	AmB
***Candida bracarensis***			
MIC_100_	100		
MFC_100_	100		
IC_50_	32.3		
***Candida glabrata***			
MIC_100_	200		
MFC_100_	200		
IC_50_	18.7		
***Candida guilliermondii***			
MIC_100_	50		
MFC_100_	50		
IC_50_	11.5		
***Candida krusei***			
MIC_100_	25	>200	1.56
MFC_100_	50	nd	1.56
IC_50_	5.0	91.29	0.46
***Candida nivariensis***			
MIC_100_	100		
MFC_100_	200		
IC_50_	54.2		

a MIC_100_ was determined as the lowest concentration of the studied peptide that inhibited 100% fungal growth. Data are representative of two independent experiments.

b MFC_100_ was determined as the lowest concentration of the studied peptide that kill 100% fungal growth. Data are representative of two independent experiments.

c IC_50_ was determined as the concentration of the studied peptide that inhibited 50% fungal growth and was estimated by nonlinear regression analysis. nd: not determined. *Ca*Def2.1_G27‑K44_ (CDF-GK); Fluconazole (FLZ); Amphotericin B (AmB).

The MIC_100_ does not distinguish between the fungicidal and fungistatic effects of CDF-GK. To differentiate between these effects, the test cells were washed with Sabouraud medium to remove the peptide and then cultured in fresh medium without the compound. The minimum fungicidal concentration (MFC_100_) of CDF-GK was determined for all tested yeasts, revealing that the inhibitory effect of CDF-GK is fungicidal, which is a highly desirable mode of action. The lowest effective concentrations (50 μM) were observed for *C. krusei* and *C. guilliermondii* (Table 1).

Given that CDF-GK exhibited the highest antifungal activity against *C. krusei* among the tested species, we compared its efficacy with that of the main antifungal agents used in clinical practice: fluconazole (FLZ) and amphotericin B (AmB) (Table 1). CDF-GK was more effective than FLZ, with MIC_100_ and IC_50_ values being 8 and 18 times lower, respectively. However, AmB demonstrated greater efficacy than CDF-GK, with MIC_100_ and IC_50_ values being 16 and 10.8 times lower, respectively.

### Time-Kill Kinetics of CDF-GK against *C. krusei*

After determining MFC_100_, we sought to establish the minimum time required for the CDF-GK peptide to induce a loss of viability in *C. krusei* cells. Our results showed that CDF-GK, at a concentration of 50 μM, causes a significant reduction in colony formation observed at 0 and 0.5 h, where only six and one CFU were detected, respectively. After 1 h, CDF-GK completely eliminated the viability of *C. krusei* cells (Figures 2 and S1). These findings indicate that CDF-GK exerts its lethal effect on *C. krusei* within the first few minutes of incubation.

**Figure 2:**
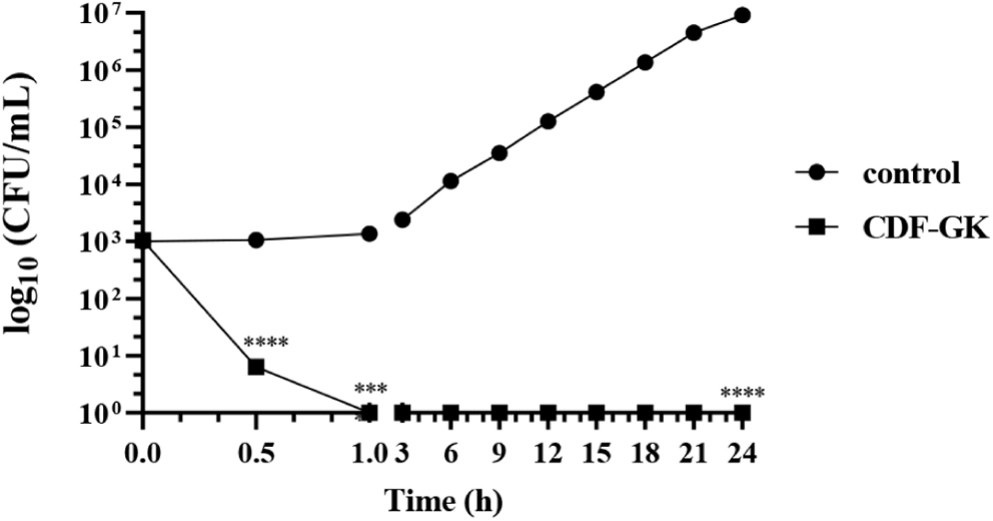
Time-kill kinetics of CDF-GK against *C. krusei*. Log_10_ CFU/mL reduction over time after treatment with 50 μM CDF-GK. Data represent mean ± SD (*n* = 3). Statistical significance was determined by one-way ANOVA (****p < 0.0001). Representative plate images are provided in Figure S1.

### *C. krusei* Biofilm Formation Inhibition Assay

The ability of CDF-GK to inhibit *C. krusei* biofilm formation was evaluated at various concentrations: MIC, 2 × MIC, 4 × MIC, and 6 × MIC (Figure 3a). Optical microscopy images revealed heterogeneous biofilm structures with dense cellular aggregates but no hyphal dominance. The results showed that CDF-GK significantly reduced biofilm formation at all tested concentrations compared to the control. At 2 × MIC, 4 × MIC, and 6 × MIC, CDF-GK completely inhibited biofilm formation (100%). Even at MIC, CDF-GK reduced biofilm formation by 48%, as confirmed by microscopy images (Figure 3c and d). In comparison, AmB treatment also significantly inhibited biofilm formation, achieving complete inhibition at 2 × MIC, 4 × MIC, and 6 × MIC. However, at MIC, AmB inhibited 86% of biofilm formation, nearly double the inhibition achieved by CDF-GK at MIC (Figure 3b).

**Figure 3:**
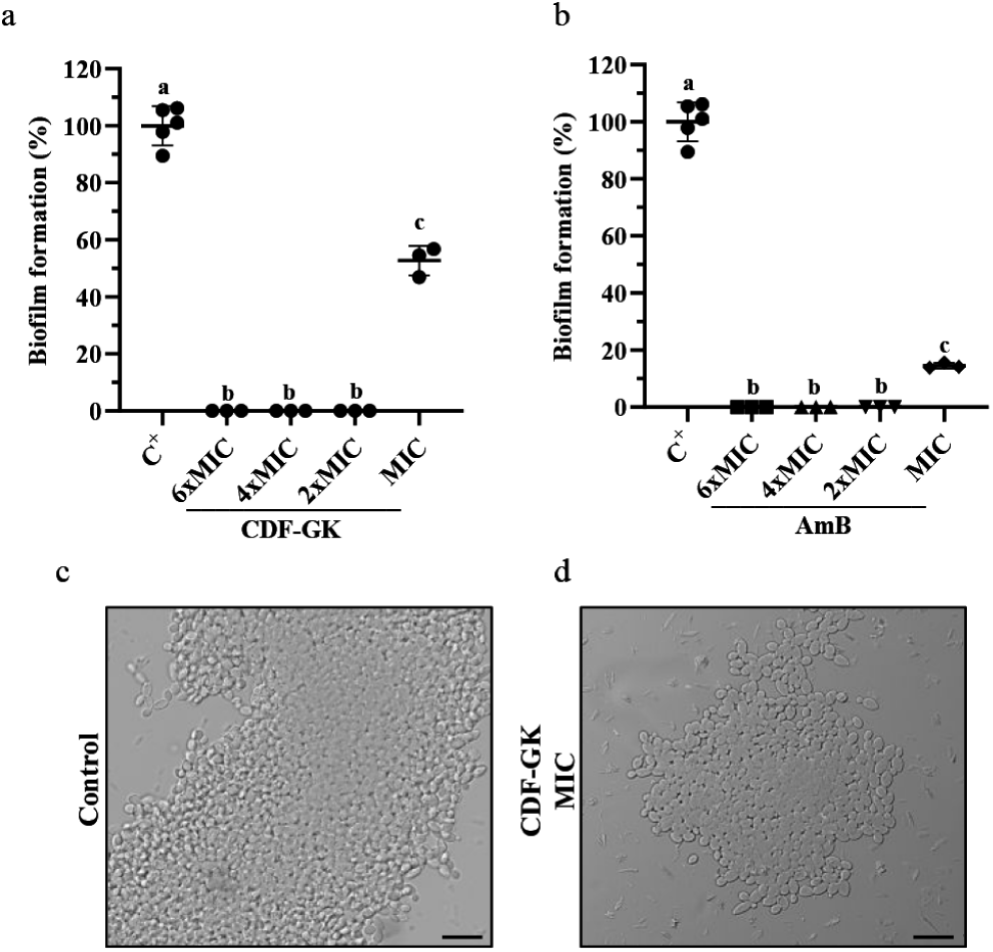
Effect of CDF-GK (a) and amphotericin B (b) on *C. krusei* biofilm formation at concentrations equivalent to MIC, 2 × MIC, 4 × MIC, and 6 × MIC previously determined for planktonic cells. Data represent mean ± SD (bars) with individual replicates (scatter points; *n* = 3) The assay is representative of an independent assay out of three. Different letters denote statistical differences (*p* < 0.05). Optical microscopy images of the control (c) and CDF-GK-treated (d) *C. krusei* biofilm. Scale bars: 20 μm.

### Membrane Permeabilization

The uptake of Sytox Green by *C. krusei* cells treated with 5 μM CDF-GK (Figure 4) suggests plasma membrane permeabilization, a mechanism likely contributing to the peptide’s fungicidal activity. CDF-GK appears to induce membrane permeability, significantly reducing the cell count and triggering pseudohyphal formation (Figure 4). A similar effect was observed in the positive control, where cells were treated with the detergent Triton X-100. It must be emphasized that the comparison of control and peptide-treated samples within the same time point can be interpreted relative to one another because the images were acquired with the same fluorescent intensity and exposure time settings, adjusted to their respective positive controls.

**Figure 4:**
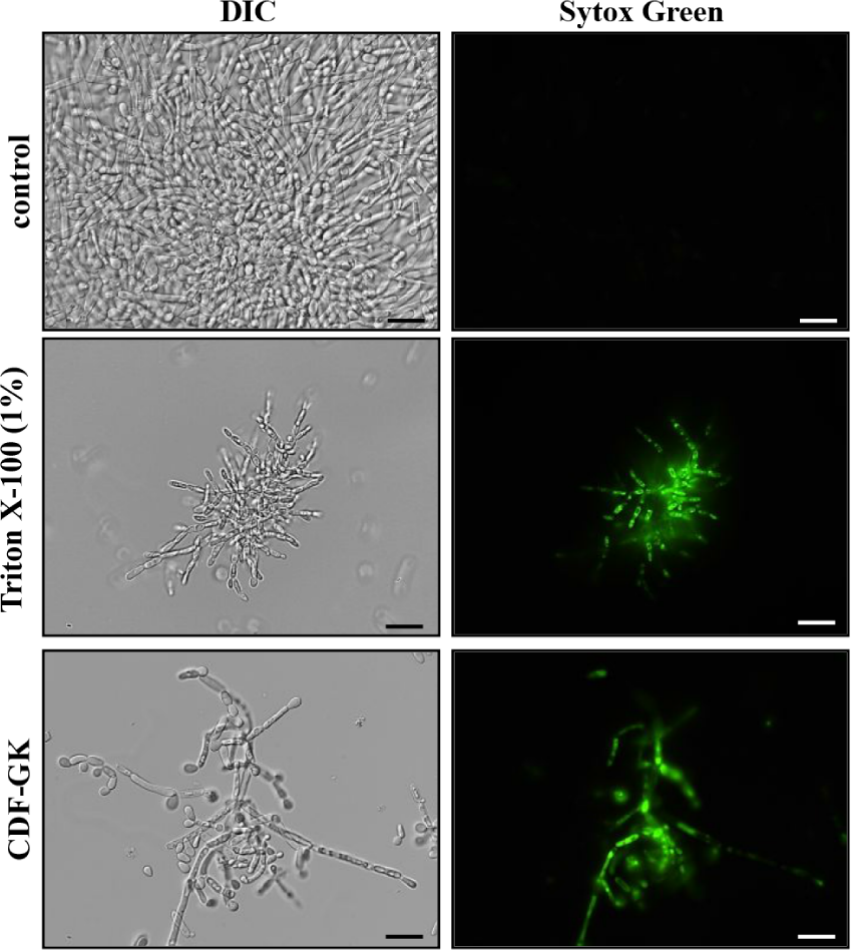
Fluorescence optical microscopy images of *C. krusei* cells after a membrane permeabilization assay using the fluorescent probe Sytox Green. Cells were treated with 5 μM CDF-GK for 24 h and then tested for membrane permeabilization. Control cells were treated with Sytox Green only, and positive control cells were treated with 1% Triton X-100. Scale bars: 20 μm.

### Secondary Structure of CDF-GK in Fungal and Mammalian Membrane Mimics

Figure 5a shows the CD spectra of CDF-GK in the absence and presence of model membranes mimicking *Candida* genus and mammalian plasma membranes. In an aqueous solution, CDF-GK displays a characteristic negative band near 198 nm, indicating a predominantly disordered structure. Upon interaction with the mammalian model membrane (POPC/SM/Chol), the negative peak shifts from 198 to 204 nm and becomes more negative, suggesting the acquisition of a more ordered conformation. In contrast, the CD spectra of CDF-GK in the presence of fungal-like membranes containing ergosterol (POPC/POPS/Erg and POPC/POPE/POPS/Erg) reveal two prominent negative bands at 209 and 224 nm along with a positive band near 195 nm. These features are characteristic of an α-helical secondary structure (Figure 5a). A similar, though less intense, helical signature is observed in the spectrum obtained in the presence of the PC/PE/PS membrane, indicating partial α-helical folding. Replacing ergosterol with cholesterol in the POPC/POPE/POPS/Erg membranes significantly reduces the intensities of the α-helical bands, indicating a lower α-helical content. This observation highlights the critical role of ergosterol in stabilizing the peptide’s secondary structure and underscores the negative impact of cholesterol on α-helix formation in these lipid environments. The calculated fraction of α-helical content, determined through spectral deconvolution using DichroWeb, confirms a significantly higher helical fraction for CDF-GK in ergosterol-containing membranes compared to both the cholesterol-containing mammalian-like membranes and the ergosterol-free fungal-like membrane (Table 2). This finding underscores the structural dependence of CDF-GK on the specific lipid composition of the membrane, particularly highlighting the essential role of ergosterol in stabilizing and promoting α-helix formation.

**Figure 5:**
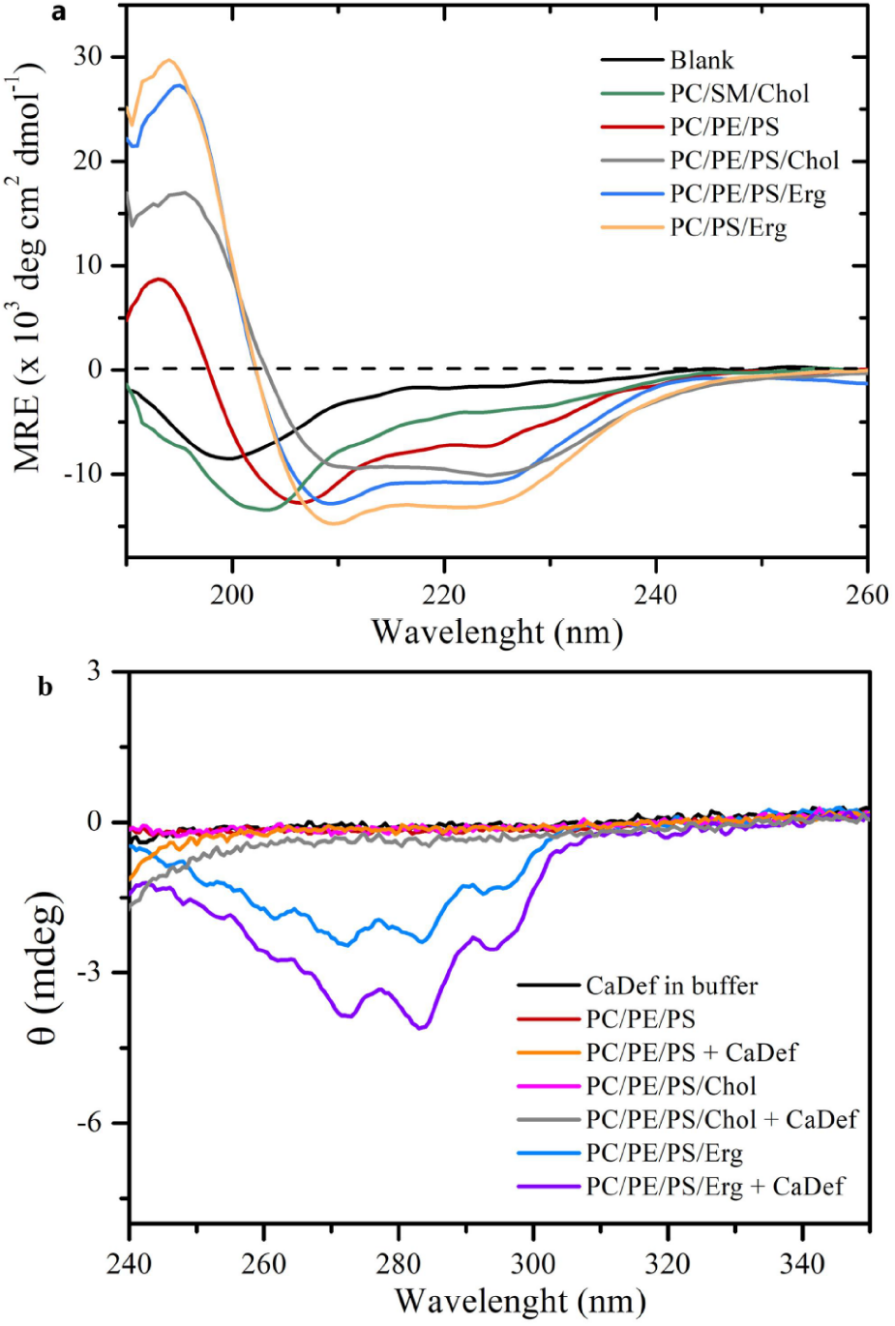
Secondary structure of CDF-GK in membrane mimics. (a) CD spectra of 30 μM CDF-GK with and without 450 μM SUVs composed of POPC/POPE/POPS, POPC/POPE/POPS/Erg, POPC/POPS/Erg, POPC/POPE/POPS/Chol, and POPC/SM/Chol. Data were recorded at 30 °C and converted to mean residue ellipticity (MRE). (b) Far-UV CD spectra of 450 μM of various SUVs in the absence and presence of 30 μM CDF-GK. Data were recorded at 30 °C and displayed in millidegrees. Buffer: 5 mM HEPES, pH 7.0.

**Table 2:** Secondary Structure Fractions Calculated from Spectral Deconvolution Using DichroWeb^a^

Sample	*T* (°C)	NRMSD	α	β	*T*	*U*	*Data set*
Blank	30	0.033	0.07	0.28	0.29	0.34	4
Blank	37	0.030	0.09	0.30	0.25	0.35	4
PC/SM/Chol	30	0.036	0.17	0.24	0.28	0.32	4
PC/SM/Chol	37	0.036	0.13	0.29	0.25	0.34	4
PC/PE/PS	30	0.030	0.32	0.18	0.24	0.25	4
PC/PE/PS/Chol	30	0.028	0.45	0.17	0.15	0.25	7
PC/PE/PS/Erg	30	0.011	0.54	0.18	0.11	0.17	4
PC/PS/Erg	30	0.013	0.58	0.14	0.12	0.15	4

a The best solutions, indicated by the lowest NRMSD values, were obtained using the CDSSTR Method along with data sets 4 and 7.

Additionally, the negative peaks at 272, 283, and 294 nm observed exclusively in the CD spectra of membranes containing ergosterol are likely associated with the electronic transitions of the ergosterol molecule (Figure 5b). The absence of these peaks in membranes lacking ergosterol further supports the notion that ergosterol is the primary contributor to the observed spectral features. The enhancement of these negative peaks upon peptide binding suggests a specific interaction between the peptides and ergosterol-containing membranes. This interaction may induce local reorganization or clustering of ergosterol molecules, altering their average orientation and resulting in enhanced chiral optical activity. These findings underscore the role of ergosterol in mediating peptide-membrane interactions and stabilizing peptide-induced membrane structures.

### Increased Endogenous ROS Production

Endogenous ROS production was analyzed using the probe 2’,7’-dichlorofluorescein diacetate (H_2_DCFDA). This dye passively enters the cell, where it is deacetylated by intracellular esterases and becomes fluorescent upon oxidation by ROS. Figure 6 illustrates the increase in ROS production in *C. krusei* cells, indicating increased oxidative stress following incubation with 5 μM CDF-GK. No fluorescence was detected in the control group (without peptide), whereas fluorescent cells were observed in the positive control (3% acetic acid).

**Figure 6:**
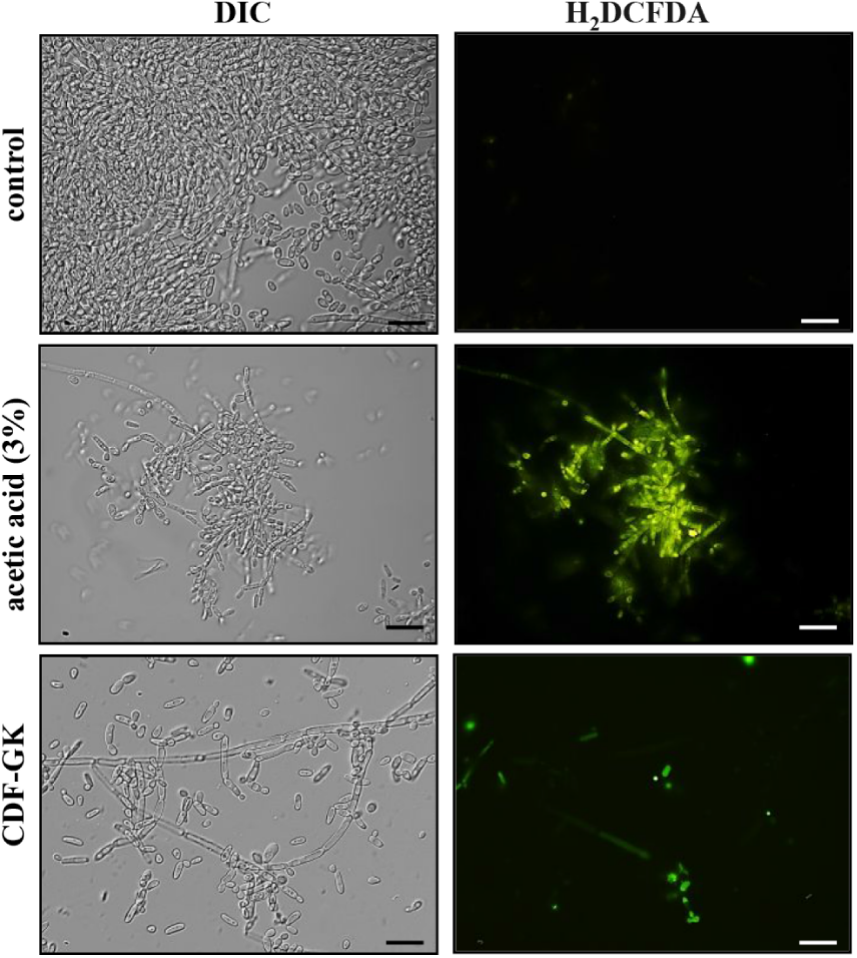
Fluorescence optical microscopy images of *C. krusei* cells after a ROS induction assay using the fluorescent probe H_2_DCFDA. Cells were treated with 5 μM CDF-GK for 24 h and then tested for oxidative stress. Control cells were treated with H_2_DCFDA only, and positive control cells were treated with 3% CH_3_COOH (acetic acid). Scale bars: 20 μm.

### Mitochondrial Membrane Potential (Δψ_m_)

The mitochondrial membrane potential (Δψ_m_) of *C. krusei* cells under control conditions and after treatment with CDF-GK was assessed via the JC-1 probe. JC-1 is a cationic, lipophilic dye that can permeate the plasma membrane. In cells with a high Δψ_m_, the dye forms aggregates within the mitochondrial matrix (J-aggregates) and emits red fluorescence. Conversely, in cells with a low Δψ_m_, the dye remains in its monomeric form in the cytoplasm, emitting green fluorescence. These observations indicate that CDF-GK induces mitochondrial depolarization, as evidenced by the reduced formation of J-aggregates, leading to decreased red fluorescence and increased green fluorescence intensity. A similar effect was observed in positive control cells treated with Triton X-100. In contrast, the negative control cells presented a greater Δψ_m_, as indicated by the pronounced red shift of the probe (Figure 7).

**Figure 7:**
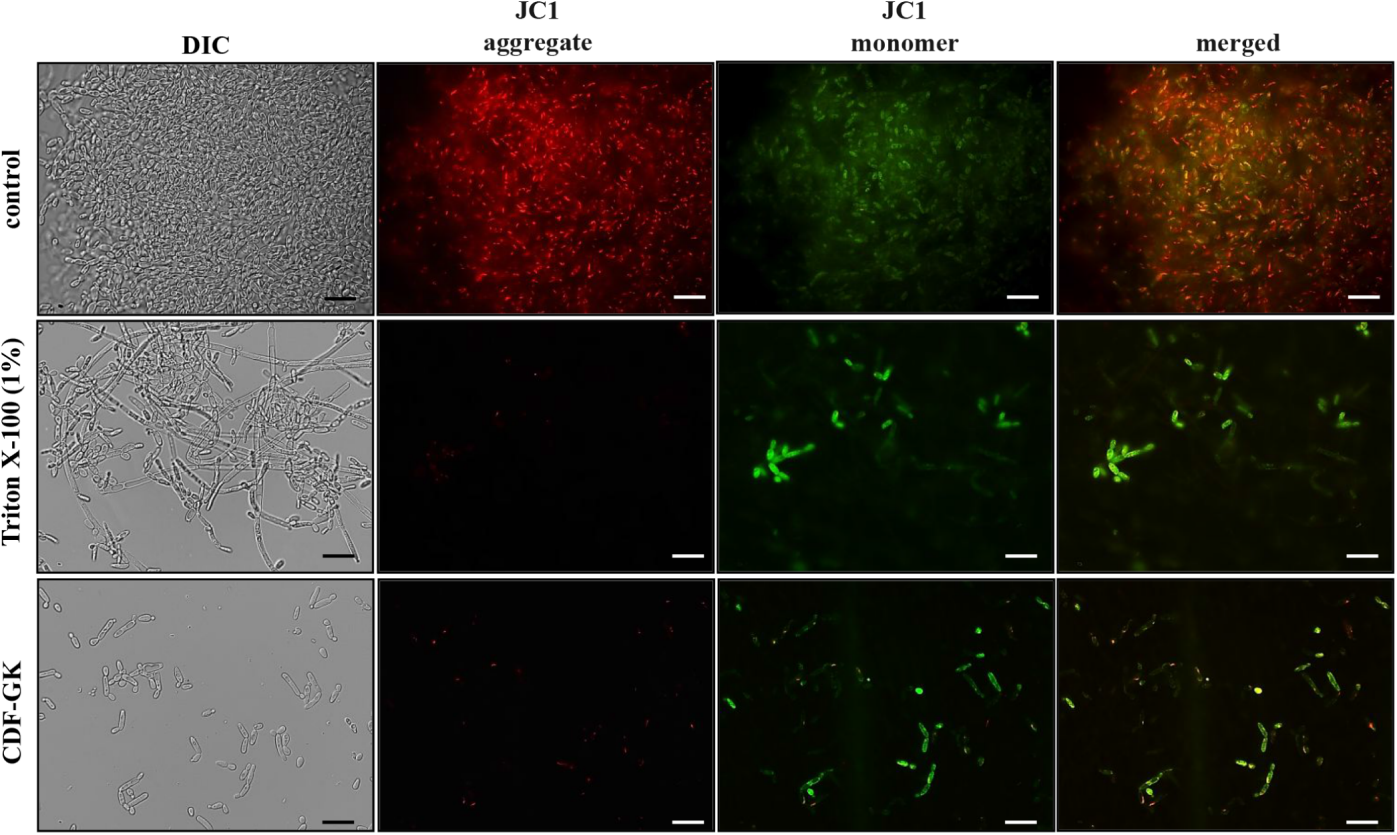
Effect of CDF-GK on the mitochondrial membrane potential of *C. krusei*. Untreated (control) cells or cells treated with 5 μM CDF-GK for 24 h. Cells treated with Triton X-100 were used as positive control. JC-1, a cationic dye, accumulates as J-aggregates (red) in cells with normal mitochondrial membrane potential, and after depolarization, it remains as a monomer, emitting green fluorescence. Scale bars: 20 μm.

### Vacuolar Membrane Fragmentation

After incubation for 60 min at 30 °C, the FM4-64 probe clearly stained the vacuole membrane, showing a ring staining pattern in practically all of the cells in the control group. In contrast, in cells treated with CDF-GK, a distinct staining pattern was observed compared to that in the control cells. In this case, a dotted marking stands out, suggesting that CDF-GK may impact the integrity and structure of the vacuolar compartment (Figure 8).

**Figure 8:**
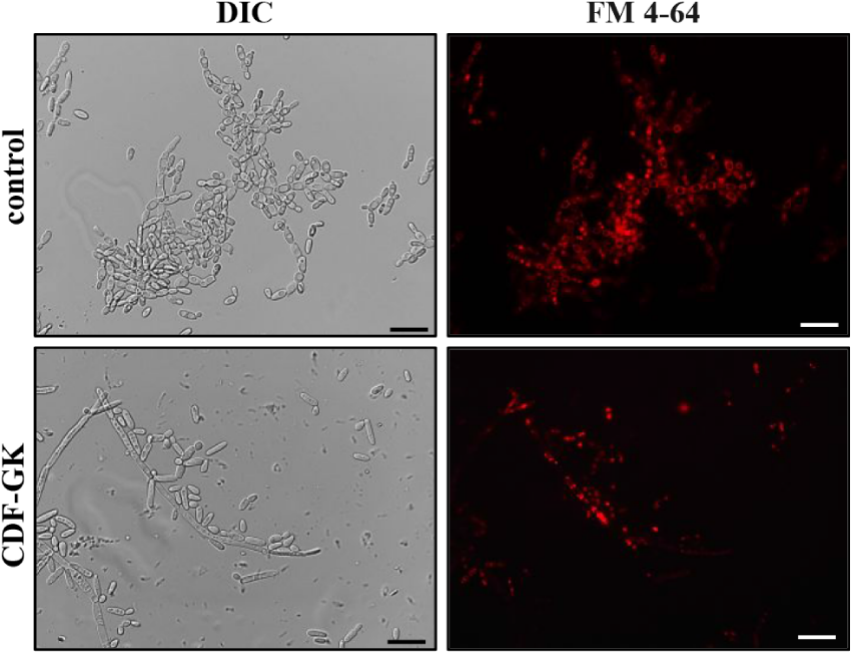
Vacuolar mapping was performed using the FM4-64 probe. Cells were treated with 5 μM CDF-GK for 24 h and then incubated with FM4-64 for 60 min at 30 °C. The control was treated with FM4-64 only. Scale bars: 20 μm.

### Kinetics of CDF-GK Entry into *C. krusei* Cells

Before conducting the time-lapse assay with the peptide coupled to 5-carboxyfluorescein (5-FAM), its antifungal activity was validated through an MFC_100_ (50 μM) assay against *C. krusei*. The results showed no significant difference in activity between 5-FAM-CDF-GK and the unconjugated form (Figure S2), confirming the preservation of its antifungal properties. After validation, we used confocal fluorescence microscopy with the peptide coupled to 5-FAM to investigate the interaction of CDF-GK with *C. krusei* cells and its ability to enter the intracellular space at a fungicidal concentration (50 μM). After 20.5 min of incubation with 5-FAM-CDF-GK, we observed intense green fluorescence staining in the intracellular space of *C. krusei* cells, indicating the entry of the peptide (Figure 9). To evaluate real-time entry kinetics, we monitored 5-FAM-CDF-GK fluorescence over time in *C. krusei* cells. The results revealed that at time 0, only the cell wall stained with Calcofluor White (fluorescent blue) was visible, indicating that the peptide had not yet reached the intracellular space. From 3.5 min onward, we observed green fluorescent labeling in the intracellular space, suggesting that this would be the minimum time for the beginning of peptide entry. The accumulation of 5-FAM-CDF-GK within the cell progressively increased with the incubation time, which was accompanied by an increase in fluorescence intensity. The maximum fluorescence peak was reached at 20.5 min.

**Figure 9:**
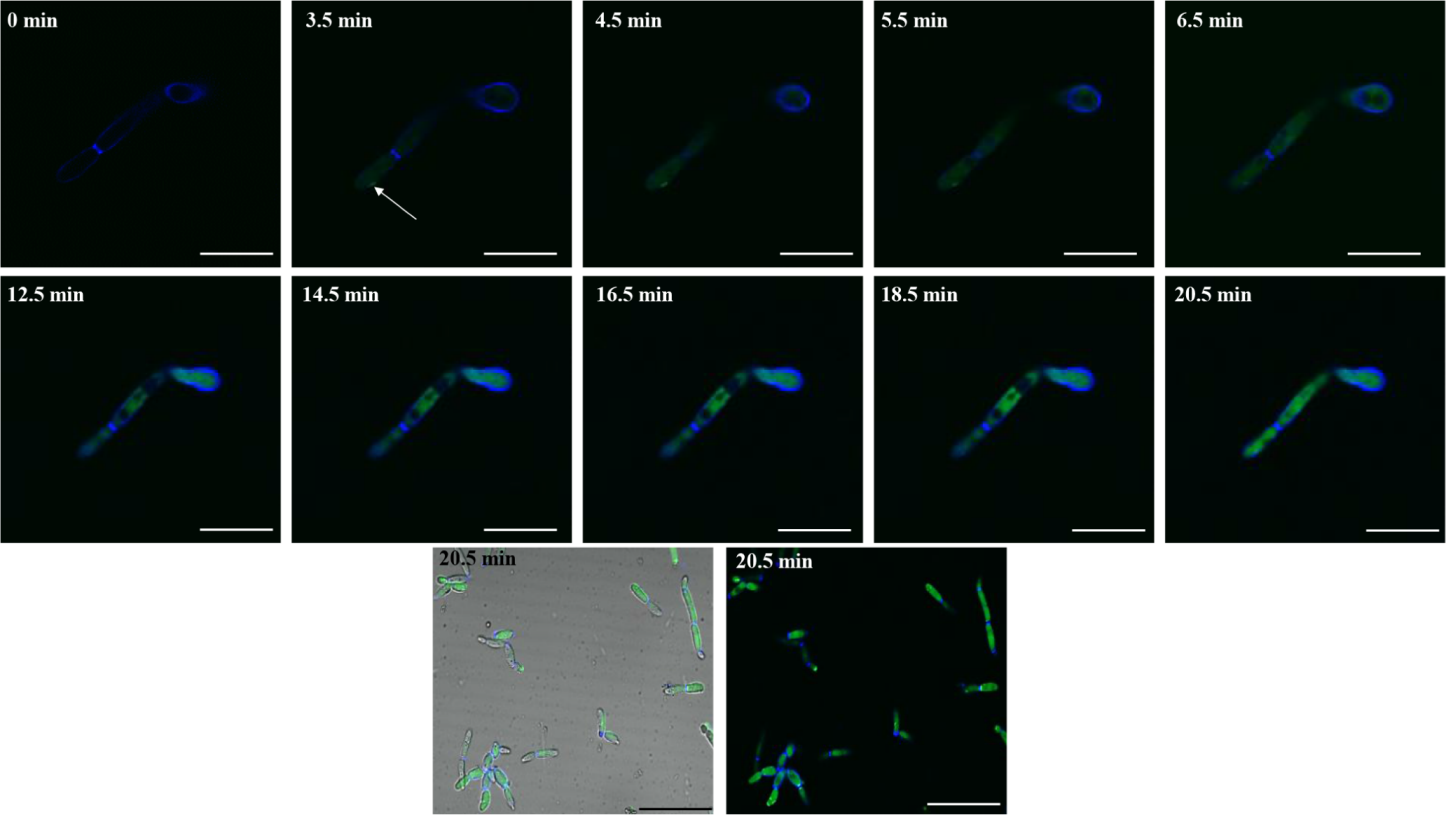
Time-lapse confocal microscopy showing the entry of 5-FAM-CDF-GK (50 μM) into *C. krusei* cells. At time 0, only the cell wall stained with Calcofluor White (blue fluorescence) is visible. From 3.5 min onward, green fluorescence (5-FAM-CDF-GK) is observed inside the intracellular space (arrow). The fluorescence intensity peak was reached at 20.5 min. Scale bars: 10 μm.

### Evaluation of CDF-GK Toxicity and Therapeutic Potential *In Vivo*

To evaluate the toxicity of CDF-GK *in vivo*, we used *G. mellonella* larvae (Figure 10a). No significant toxic effects were observed in larvae inoculated with high concentrations of CDF-GK (1000, 500, and 250 μM) compared with the control groups, which included larvae subjected only to mechanical injury from the syringe and those injected with PBS.

**Figure 10:**
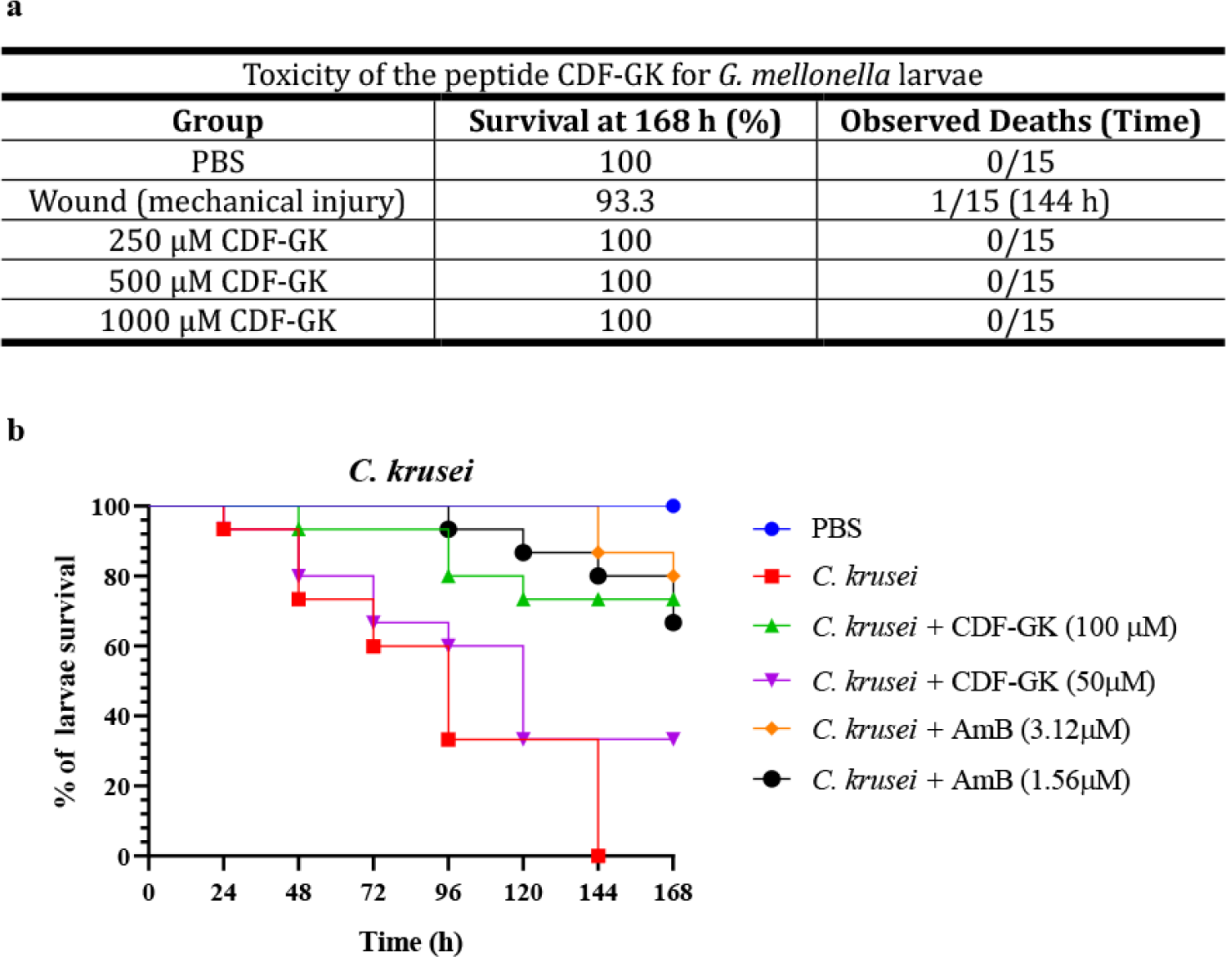
Toxicity and therapeutic activity of CDF-GK. (a) Toxicity of the peptide CDF-GK for *G. mellonella* larvae. The larvae were treated with concentrations of CDF-GK ranging from 250 to 1000 μM. Wound refers to damage caused only by the injection needle. (b) Survival curves of *G. mellonella* larvae infected with *C. krusei* (10^6^ cells/larva) and treated with CDF-GK at concentrations of 50 and 100 μM or Amphotericin B at 1.56 and 3.12 μM. PBS indicates that larvae were inoculated only with phosphate-buffered saline. *C. krusei* represents larvae infected with 10^6^ cells/larva and untreated. Results are the mean of three independent experiments. Statistical significance was determined using the Gehan–Breslow–Wilcoxon test, *p* ≤ 0.05.

To assess the therapeutic potential of CDF-GK, an *in vivo* assay was conducted using *Galleria mellonella* larvae infected with *C. krusei*. Initially, larvae infected with *C. krusei* were used to determine the minimum lethal concentration (MLC). Inocula ranging from 10^3^ to 10^7^ CFU/larva were tested, and the MLC was identified at 10^6^ CFU/larva (data not shown). On the basis of these findings, a concentration of 1 × 10^6^ CFU/larva was selected to evaluate the effect of CDF-GK on experimental candidiasis. Treatment of larvae infected with *C. krusei* via CDF-GK at concentrations of 50 and 100 μM, corresponding to the minimum fungicidal concentration (MFC) and twice the MFC *in vitro*, respectively, resulted in a significant increase in the survival rate (Figure 10b). CDF-GK treatment extended the survival of infected larvae to 168 h. The survival rates were 73% and 33% with 100 and 50 μM CDF-GK, respectively (Figure 10b). In comparison, treatment with AmB provided protection rates of 66.6% and 80% at concentrations equivalent to those of MFC (1.56 μM) and twice those of MFC (3.12 μM) *in vitro*, respectively. Although AmB demonstrated greater efficacy at lower concentrations, these results suggest that CDF-GK is a potent molecule with protective effects comparable to those of commercial antifungals.

### Melanization Quantification and Determination of Hemocyte Density

We observed a rapid onset of melanization in larvae within 3 h post-infection with *C. krusei* when the larvae were treated with only PBS (*C.k* + PBS). This melanization intensified significantly after 24 h, with a substantial accumulation of melanin in the hemolymph of infected and untreated larvae (*C.k.* + PBS), which presented levels approximately 3.5 times higher than those in larvae treated with CDF-GK (*C.k.* + CDF-GK) (Figure 11a). No significant difference in melanization was observed between uninfected larvae (PBS) and those infected with 100 μM CDF-GK (*C.k.* + CDF-GK) at any of the tested time points (Figure 11a). These findings suggest that CDF-GK effectively protected infected larvae, potentially by inhibiting *C. krusei* proliferation in the hemolymph, thereby preventing the activation of the melanization process for up to 24 h, as illustrated in the images of treated and untreated larvae. In untreated larvae, more pronounced melanization was observed in the dorsal region (Figure 11b).

**Figure 11:**
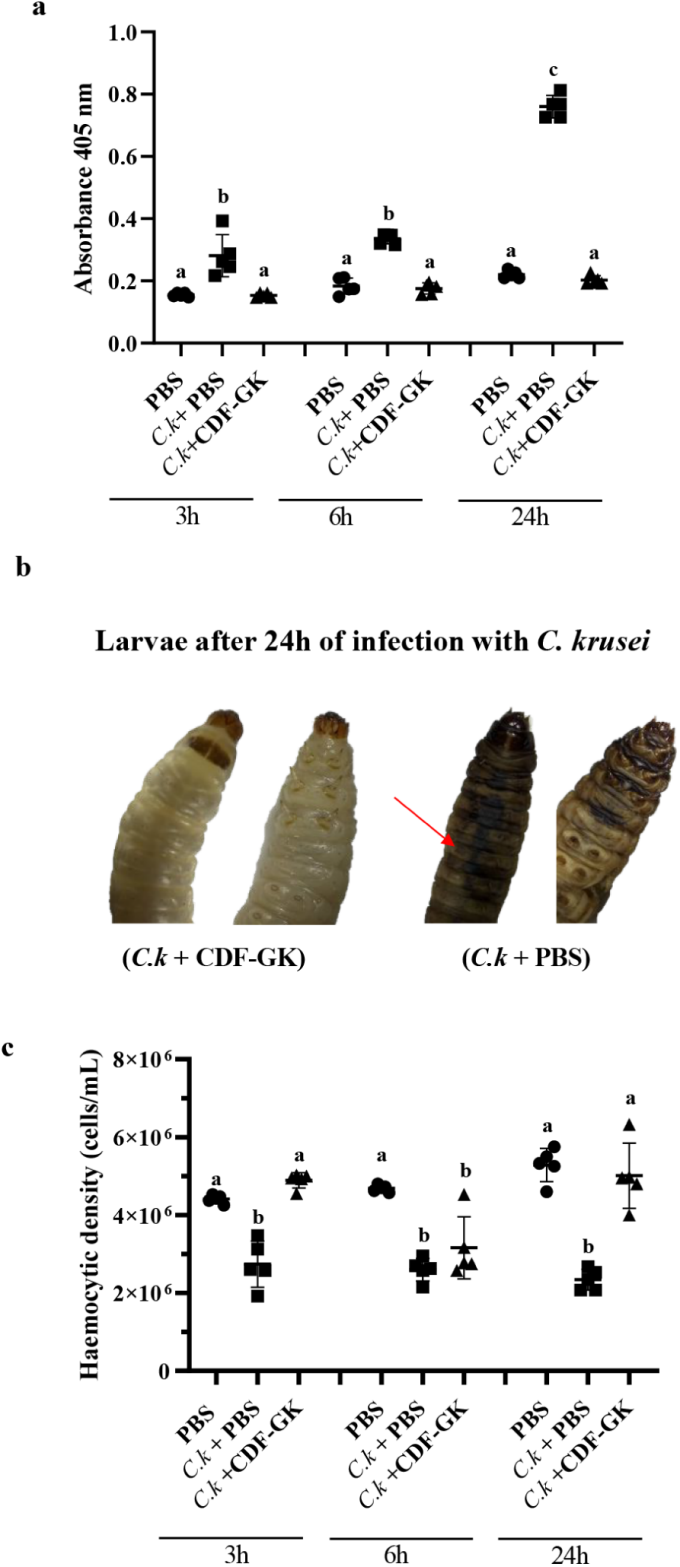
Melanization and hemocyte density in *G. mellonella* infected with *C. krusei*. (a) Optical density of hemolymph from *G. mellonella* collected 3, 6, and 24 h after infection with 10^6^ cells/larva of *C. krusei*. Groups include larvae injected with PBS (PBS); larvae inoculated with 10^6^ *C. krusei* cells/larva and treated with PBS (*C.k* + PBS); and larvae inoculated with 10^6^ *C. krusei* cells/larva and treated with 100 μM CDF-GK (*C.k* + CDF-GK). (b) Images of larvae at 24 h showing prominent melanization in the dorsal vessel (arrow) in the control group compared with those treated with CDF-GK. (c) Hemocyte density in the hemolymph of *G. mellonella* larvae after 3, 6, and 24 h of infection with 10^6^ cells/larva of *C. krusei* or inoculated with PBS, estimated using a hemocytometer. The groups are the same as those described above. All larvae were incubated at 37 °C Different letters indicate significant differences, while the same letter indicates no difference (*p* < 0.05). Data represent the mean ± SD (bars) with individual replicates (scatter points; *n* = 5). The assay is representative of an independent assay out of two.

We also assessed the hemocyte concentration in larvae infected with *C. krusei* and treated with or without 100 μM CDF-GK. The results revealed a 2.3-fold decrease in hemocyte density in *C. krusei*-infected larvae compared with that in uninfected controls (PBS) and larvae treated with CDF-GK (Figure 11c). Although the hemocyte density in CDF-GK-treated larvae decreased similarly to that in untreated larvae at 6 h postinfection, it recovered to levels comparable to those in uninfected larvae by 24 h postinfection. Together, the melanization and hemocyte density data underscore the protective effect of CDF-GK in *C. krusei*-infected larvae.

## Discussion

The treatment of fungal infections remains a significant challenge for global healthcare, mainly because of the limited availability of antifungal agents that combine low toxicity to nontarget organisms with high clinical efficacy. Expanding the current antifungal repertoire by identifying new therapeutic targets and developing innovative strategies is crucial to overcoming these limitations. In 2022, the World Health Organization (WHO) emphasized this need by identifying several *Candida* species as high-priority pathogens, classified across all risk categories, from critical to medium risk.^34^

In this study, we evaluated the antimicrobial properties of a rationally designed peptide, *Ca*Def2.1_G27‑K44_ (CDF-GK), derived from the defensin *Ca*Def2.1, which was originally isolated from *Capsicum annuum* fruits.^35^ This peptide, consisting of 18 amino acids, was designed by Taveira et al.^19^ and tested against non-*albicans Candida* (NAC) species. Our investigation explored its antifungal mechanism, cytotoxicity, and therapeutic potential *in vivo* using *Galleria mellonella* larvae as an infection model.

Our initial tests demonstrated that CDF-GK exhibited significant antifungal activity against all of the tested *Candida* species. However, the inhibitory and fungicidal effects were particularly potent against *C. krusei* (renamed *Pichia kudriavzevii*), with the lowest MIC_100_, MFC_100_, and IC_50_ values recorded at 25, 50, and 5 μM, respectively (Table 1). These findings are consistent with those reported by Souza et al.,^36^ who designed a peptide named JcTI–PepI, bioinspired by the primary structure of a trypsin inhibitor purified from *Jatropha curcas* seeds. Like CDF-GK, JcTI-PepI exhibited antifungal activity against all of the tested *Candida* strains, with the most potent inhibitory action also observed against *C. krusei*, where the MIC (31.25 μM) and MFC (62.5 μM) values were comparable to those reported for CDF-GK against the same *C. krusei* strain (ATCC 6258).

*Candida krusei* has emerged as a significant concern in clinical settings, particularly because of its intrinsic resistance to fluconazole (FLZ), a commonly used antifungal agent. This resistance, often exacerbated by the prophylactic or therapeutic use of FLZ, complicates the management of candidiasis.^16^ The WHO classified *C. krusei* as a medium-risk fungal pathogen in 2022, highlighting the need for alternative treatments.^34^

In our study, the peptide CDF-GK demonstrated superior inhibitory activity against *C. krusei* compared with FLZ. Specifically, CDF-GK was 18 times more effective in reducing 50% of the fungal cells (IC_50_) than FLZ (Table 1). However, while amphotericin B (AmB) has greater efficacy, with an IC_50_ 10.8 times lower than that of CDF-GK, the known toxicity and inconsistent effectiveness of AmB emphasize the ongoing need for safer, more reliable antifungal agents.^37–39^

In addition to its inhibitory properties, CDF-GK exhibited rapid fungicidal action, killing 99% of the *C. krusei* cells within 30 min and achieving complete cell death within 1 h (Figure 2). This rapid action is critical for reducing the opportunity for resistant strains to develop.^40^ Comparable kinetics have been reported for other bioinspired peptides. For example, Lucas et al.^41^ demonstrated that the WR peptide derived from *Vigna unguiculata* defensin *Vu*Def_1_ eliminated 97% of *C. albicans* cells immediately and 100% within 1 h. This finding matches the performance of CDF-GK against *C. krusei* and reinforces the relevance of rapid-acting peptides in antifungal therapy. The rapid killing and significant activity against fluconazole-resistant species underscore the therapeutic potential of CDF-GK and the need for further exploration in *in vivo* models, particularly given the limitations of current antifungal treatments.

*Candida* species are well-documented for their ability to form complex, structured biofilms that include various morphological forms.^42^ Biofilms are implicated in approximately 80% of human microbial infections, as reported by the National Institutes of Health.^43^ The biofilm matrix provides a protective environment that enhances fungal survival and shields it from host immune responses, often leading to increased drug resistance.^44,45^ The ability of *C. krusei* to form biofilms on inert surfaces, such as catheters and prosthetic devices, is a key virulence factor contributing to its pathogenicity. These biofilm-covered surfaces can act as entry points for bloodstream infections in hospitalized patients, significantly increasing the risk of candidaemia. Additionally, biofilms exhibit resistance to antifungal agents at concentrations much higher than those required to inhibit planktonic cells.^46^

Given the potent and rapid fungicidal activity of CDF-GK, we further investigated its efficacy in inhibiting *C. krusei* biofilm formation. Our results show that CDF-GK significantly inhibited biofilm formation at concentrations similar to those effective against planktonic cells, achieving complete inhibition at 2 × MIC. Comparable results were observed with AmB at 1.56 μM (2 × MIC) (Figure 3). These findings are consistent with previous studies on bioinspired AMPs. For example, PEP-IA18, a synthetic AMP inspired by the primary structure of profilin from *Spodoptera frugiperda* (fall armyworm), exhibited similar effects on the inhibition of biofilm formation by *C. albicans* and *C. tropicalis*. At the MIC (2.5 μM) and 10 × MIC (25 μM), PEP-IA18 significantly reduced biofilm formation and disrupted preformed biofilms in both species.^47^ Conversely, while the peptide JcTI-PepI was effective against planktonic yeast cells, it failed to prevent the formation of biofilms by *C. krusei*. However, a concentration of 62.5 μM JcTI-PepI reduced preformed *C. krusei* biofilms by 62%.^36^ The effect of CDF-GK on preformed biofilms remains to be tested.

Together with the literature, our findings suggest that CDF-GK, like other bioinspired AMPs, holds substantial potential as a therapeutic agent for treating infections caused by planktonic cells. Additionally, CDF-GK has demonstrated efficacy in preventing biofilm formation, which is critical for managing invasive infections caused by *C. krusei*. This is particularly significant given that *C. krusei* is intrinsically resistant to FLZ and exhibits variable sensitivity to other antifungal agents, such as AmB, voriconazole, itraconazole, posaconazole, anidulafungin, micafungin, and 5-flucytosine.^39,48^

While many AMPs are recognized for their membrane-targeting activity, the precise mechanisms by which these peptides interact with cellular membranes remain incompletely understood. The prevailing model suggests that AMPs initially engage with microbial membranes through electrostatic interactions, which are facilitated by the charge differences between the peptides and the microbial membranes. Following this initial interaction, membrane disruption or peptide internalization into the cell may occur.^49^ Previous studies by Taveira et al.^19^ demonstrated that the CDF-GK peptide can permeabilize the plasma membrane of various *Candida* species, including *C. albicans*, *C. tropicalis*, *C. buinensis*, and *C. parapsilosis*. In the present study, we confirmed this membrane-disrupting effect, showing that CDF-GK also induces damage to the cytoplasmic membrane of *C. krusei* (Figure 4). Several studies have corroborated the ability of both natural and bioinspired AMPs to increase the permeability of fungal cytoplasmic membranes.^19,41,50,51^ A recent example is the KWI-19 peptide, which directly targets the cell membrane of *C. tropicalis* by interacting with ergosterol.^52^

The composition of sterols in cell membranes is a key difference between mammals and fungi. While cholesterol (CHL) predominates in mammalian membranes, ergosterol (ERG) is the main sterol in fungi. Both sterols modulate properties such as lipid fluidity and packing, but they do so in distinct ways.^53,54^ This distinction is essential for the selectivity of antimicrobial peptides, such as CDF-GK, which demonstrate specific secondary conformations, including the formation of α-helices in the presence of ERG, as observed through circular dichroism (CD) spectroscopy (Figure 5). The results obtained highlight the importance of ERG as a preferential target in fungal membranes, promoting favorable structural interactions with antifungal peptides, contrary to what occurs with CHL in mammals. The selectivity observed in CDF-GK in the presence of ERG, as well as the stabilization of α-helices, is consistent with previous studies on the peptide VG16KRKP.^54^ Both findings suggest that ERG facilitates the structural reorganization of membranes, favoring peptide–membrane interactions and increasing antifungal efficacy. Additionally, CD spectroscopy data revealed that the peptide undergoes structural reorganization, adopting an α-helical conformation in membrane mimics containing ERG (Figure 5). This interaction and structural conformation likely destabilize lipid packing in fungal membrane mimics due to the peptide. The *C. krusei* membrane permeabilization by CDF-GK, observed via the Sytox Green assay (Figure 4), suggests CDF-GK’s interaction with ERG in fungal membranes. Similarly, AmB, a well-known membrane-active agent, also interacts with ERG, forming transmembrane pores that disrupt membrane integrity and induce cell lysis. Although CDF-GK interacts with ERG, its mechanism of action appears to differ significantly. The 5-FAM-CDF-GK data show that it did not remain in the membrane like AmB but was quickly internalized (Figure 9), suggesting the possibility of intracellular targets. This distinction between ERG and CHL interactions has important implications for the development of selective therapies. Peptides such as CDF-GK, which rely on the presence of ERG, show great therapeutic potential due to their selectivity, offering a strong foundation for the development of antifungals with lower toxicity to the host.

The interaction between AMPs and microbial membranes is crucial for cell death, although other mechanisms also contribute. One such mechanism is the induction of endogenous oxidative stress. Reactive oxygen species (ROS), including hydrogen peroxide (H_2_O_2_), hydroxyl radicals (OH^–^), singlet oxygen (^1^O_2_), and superoxide (O_2_^–^), are produced by all cell types.^55^ A primary source of ROS within cells is the mitochondrial respiratory chain, where they are generated as byproducts during ATP synthesis. While ROS play key roles in cellular signaling and essential physiological processes,^55,56^ excessive ROS levels disrupt redox balance, leading to cellular damage through the oxidation of proteins, lipids, carbohydrates, and DNA.^57,58^

In our study, *C. krusei* treated with CDF-GK presented a marked increase in ROS production (Figure 6), likely leading to significant disruptions in cellular processes. Additionally, mitochondrial membrane depolarization was observed following treatment with CDF-GK (Figure 7), indicating impaired energy metabolism. Mitochondrial depolarization is often linked to oxidative stress-induced damage, as ROS can trigger the opening of mitochondrial permeability transition pores, leading to cytochrome c release and a reduction in mitochondrial potential.^59–61^ Since most fungal pathogens depend on mitochondria for growth, survival, and ATP production, targeting mitochondrial function may represent a good antifungal strategy.^62^ These findings are consistent with those of Li et al.,^63^ who demonstrated that the CGA-N12 peptide, derived from human chromogranin A, induces ROS production, mitochondrial membrane potential dissipation, and cytochrome c release in *C. tropicalis*, ultimately resulting in mitochondria-dependent apoptosis.

In fungal cells, the vacuole is a multifunctional organelle essential for maintaining cellular homeostasis, regulating ion and pH balance, and degrading macromolecules.^31^ During optical microscopy assays, we observed morphological changes in *C. krusei* cells treated with CDF-GK, which are potentially linked to vacuole dynamics. Vacuolar morphology is known to undergo fission and fusion in response to environmental stress, such as oxidative stress, which promotes vacuole fission and leads to hyperfragmentation.^64,65^ To investigate whether CDF-GK influences vacuole morphology, we used the FM4-64 probe and confirmed vacuole fragmentation in *C. krusei* treated with the peptide (Figure 8). This finding is consistent with those of previous studies, such as Ogita et al.,^66^ who reported that polyene antifungals, such as amphotericin B and nystatin, cause vacuole disintegration in *Saccharomyces cerevisiae*, contributing to their fungicidal effects. Similarly, Parisi et al.^67^ reported that the plant defensin Ppdef1 from *Picramnia pentandras* causes vacuole rupture in *S. cerevisiae*. Ppdef1 was shown to rapidly enter the cytoplasm, increase ROS production, induce vacuolar fusion, and ultimately lead to plasma membrane permeabilization and vacuole rupture, causing cell death.

In our study, CDF-GK also penetrated the intracellular space of *C. krusei* within 3.5 min, with progressive accumulation in the cytoplasm over time (Figure 9). Although we did not monitor vacuole labeling over time, we hypothesize that the darker circular regions observed in the cytoplasm between 6.5 and 18.5 min represent vacuolar structures. The subsequent disappearance of these regions by 20.5 min, accompanied by homogeneous green staining of the peptide throughout the cytoplasm, suggested vacuole fragmentation. The peak cytoplasmic accumulation of CDF-GK at 20.5 min coincided with the timing of cell death, as 99% of the cells died within 30 min, as indicated by the results of the cell death kinetics assay (Figure 2). In contrast, the WMR peptide, bioinspired by the myxinidin sequence from *Myxine glutinosa*, demonstrated slower intracellular penetration in *C. parapsilosis*, with significant cytoplasmic accumulation only after 4 h of incubation.^68^ This difference in kinetics highlights the rapid action of CDF-GK, which may contribute to its potent antifungal effects.

A previous study by Taveira et al.^19^ demonstrated that CDF-GK exhibits low *in vitro* toxicity toward mammalian cells, including macrophages, monocytes, and erythrocytes, with an IC_50_ > 200 μM (the highest concentration tested) and minimal hemolysis (<20% at 200 μM), indicating a favorable safety profile in mammalian systems. Building on these findings, we calculated a conservative therapeutic index (TI) of 40, defined as the ratio of the mammalian cell IC_50_ (used in the calculation as the threshold of 200 μM) to the *C. krusei* IC_50_ (5 μM). This TI highlights the selectivity of CDF-GK for fungal cells and its translational potential. Notably, this estimate assumes the mammalian IC_50_ at the tested upper limit; the actual IC_50_ may be significantly higher, which would further increase the TI. Such selectivity, combined with rapid fungicidal action, makes CDF-GK a promising candidate for targeting drug-resistant *C. krusei* infections while minimizing off-target effects. On the basis of these findings, we explored the antifungal efficacy and nontoxic potential of this peptide using *G. mellonella* larvae as a study model. *G. mellonella* has gained popularity as an alternative model for studying virulence, pathogenesis, and antimicrobial efficacy.^69–72^ Despite the lack of antibody production, this insect’s immune system is complex and shares similarities with vertebrate innate immunity, including both cellular and humoral responses.^73^ The innate humoral response in *G. mellonella* is driven by processes such as melanization, hemolymph coagulation, the induction of reactive species, and the synthesis of antimicrobial peptides, which collectively inhibit pathogen proliferation and facilitate elimination.^74,75^ The strong correlation between results obtained from *G. mellonella* and mammalian models underscores its relevance, offering additional benefits such as low cost, rapid testing, large-scale reproduction, larvae incubation temperatures ranging from 25 to 37 °C, and various pathogen inoculation methods.^76–78^

In this study, we demonstrated that CDF-GK had no toxic effects on *G. mellonella* larvae, even at concentrations as high as 1000 μM, with a 100% survival rate observed over 168 h (Figure 10a). These findings contrast with those of Martins de Andrade et al.,^79^ who evaluated the toxicity of IbKTP-NH2, a derivative of the neuropeptide kyotorphin. Although IbKTP-NH2 exhibited potent antifungal activity against biofilms of various *Candida* species and showed no significant toxicity in *G. mellonella* larvae at concentrations between 125 and 500 μM, it caused 100% mortality at 1000 μM after 120 h. This difference highlights the enhanced safety profile of CDF-GK at higher concentrations. Maione et al.^68^ reported that the bioinspired peptide WMR did not exhibit toxicity in *G. mellonella* larvae. However, they tested concentrations 100 times lower than those used for CDF-GK and monitored survival for only 72 h. More recently, Almeida et al.^52^ demonstrated the safety of KWI-19, with no larval deaths observed at the highest concentrations tested (25 and 50 μM/L) over 72 h, further supporting the nontoxic nature of peptide-based treatments *in vivo*. These findings collectively suggest that CDF-GK is a safe molecule, even at high doses over extended periods, positioning it as a promising candidate for further therapeutic development.

Evaluating both toxicity and therapeutic efficacy *in vivo* is a crucial step in the development of new antimicrobial molecules. Given the strong *in vitro* antifungal activity of CDF-GK against *C. krusei* and its lack of toxicity in *G. mellonella* larvae, we investigated its therapeutic potential. Our findings revealed that CDF-GK significantly improved the survival rate of larvae infected with *C. krusei*, indicating its potential as an effective candidicidal agent *in vivo*. Similarly, Martins de Andrade et al.^79^ assessed the therapeutic potential of IbKTP-NH2 against candidiasis using *G. mellonella*. Larvae infected with *C. albicans* and treated with 500 μM IbKTP-NH2 presented an initial survival rate of 80% at 24 h, which decreased to 30% at 48 h and stabilized at 20% over 168 h. Maione et al.^68^ demonstrated that the peptide WMR, at a concentration of 10 μM, protected larvae infected with *C. albicans* and NAC species, increasing survival rates by approximately 30–40% over 72 h. Although we did not test *C. albicans*, limiting direct comparison, treatment of *C. krusei*-infected larvae with 50 and 100 μM CDF-GK resulted in survival increases of 33% and 73%, respectively, at 168 h (Figure 10b). These rates are notably higher than those reported for IbKTP-NH2 and WMR in similar models. Scorzoni et al.^33^ evaluated the *in vivo* efficacy of antifungal agents during infections caused by *C. albicans* and *C. krusei* via alternative models, including *G. mellonella*. Their data revealed that FLZ had no protective effect on the *C. krusei* infection. Additionally, they reported that higher concentrations of caspofungin and AmB were required for protection during *C. krusei* infection than during *C. albicans* infection. These findings support the idea that CDF-GK is as promising as clinically used antifungals, particularly in treating infections caused by drug-resistant strains such as *C. krusei*.

Other key parameters for evaluating the response of *G. mellonella* to pathogens and antimicrobial agents include melanization and quantification of circulating hemocyte density. Melanin, a toxic compound whose production is tightly regulated, represents a crucial humoral response catalyzed by the enzyme phenoloxidase. This process leads to the encapsulation of foreign particles, which serve as indicators of larval health.^33,80,81^ Melanization appears as dark spots on the cuticle, and as infection progresses, it can lead to complete melanization of the larva, particularly in the dorsal region comprising the heart, which correlates with subsequent larval death.^70,82,83^ In this study, we observed that larvae infected with *C. krusei* and treated with CDF-GK presented melanization levels comparable to those of noninfected larvae (Figure 11a). In contrast, untreated infected larvae presented a significant increase in melanization.

Another relevant parameter is hemocyte density, which plays a central role in the cellular immune response of larvae. These hemocytes, analogous to human phagocytes, are involved in processes such as phagocytosis, encapsulation, and nodule formation.^70,84,85^ In our study, we demonstrated that larvae infected with *C. krusei* and treated with CDF-GK maintained a high density of circulating hemocytes, whereas untreated infected larvae presented a drastic reduction in hemocyte count (Figure 11c). There is a direct correlation between hemocyte density and larval survival rates; infected individuals often display a lower hemocyte density than noninfected controls, reflecting greater susceptibility to infection.^84^ Additionally, the reduction in hemocyte count is also associated with the migration of these cells to infection sites, where they form nodules in response to the pathogen.^33^

On the basis of these findings, we conclude that maintaining a high density of circulating hemocytes and the absence of melanization, similar to noninfected larvae, indicates that CDF-GK is effective in controlling *C. krusei*-induced candidiasis. These results suggest that the peptide reduces the fungal burden in larvae, minimizing the need for the activation of both cellular and humoral immune responses.

## Conclusion

The findings of this study demonstrate that the bioinspired peptide CDF-GK has significant potential as an antifungal agent, particularly against *Candida* species, including non-*albicans* strains resistant to conventional treatments. Notably, CDF-GK exhibited superior efficacy compared to FLZ and comparable activity to AmB in inhibiting the growth of *C. krusei* as well as reducing cell viability and preventing biofilm formation. Mechanism of action analyses revealed that CDF-GK induces cell membrane permeabilization and intracellular damage. Furthermore, our findings highlight the crucial role of ergosterol in promoting the α-helical structure of CDF-GK and mediating its interaction with fungal membranes, suggesting its potential as a selective antifungal agent. The generation of reactive oxygen species (ROS) and mitochondrial membrane depolarization suggests that the peptide may trigger programmed cell death. Additionally, vacuolar fragmentation and peptide internalization support the hypothesis that specific intracellular targets enhance antifungal efficacy. *In vivo* tests using *G. mellonella* larvae confirmed the low toxicity of the peptide, even at high concentrations, and its ability to significantly improve the survival rate of larvae infected with *C. krusei*. Moreover, assays quantifying melanization and hemocyte density further supported the safety and efficacy of CDF-GK in an animal model. In conclusion, this study provides strong evidence that CDF-GK is a promising candidate for combating resistant fungal infections, demonstrating a favorable safety profile and potent antifungal activity in both *in vitro* and *in vivo* models.

## Supplementary Material



## Notes

Petri dish images showing the kinetics of *C. krusei* cell death over time, illustrating colony growth in control conditions and after treatment with CDF-GK. Validation of the antifungal activity of the 5-FAM-conjugated peptide, confirming that the 5-FAM labeling does not impair its antifungal properties (PDF)
