# Supplementary material for: In Vitro and In Vivo Antifungal Efficacy and Safety of the CaDef2.1G27‑K44 Peptide against the Neglected and Drug-Resistant Pathogen Candida krusei
Source: ACS Bio Med Chem Au. 2025 May 13;5(4):620–36. doi: 10.1021/acsbiomedchemau.5c00020 (PMC12371490; doi:10.1021/acsbiomedchemau.5c00020)
Supplement: Supplementary file 1 [file bg5c00020_si_001.pdf]

In vitro and *in vivo* antifungal efficacy and safety of the *Ca*Def2.1$_{G27\text{-}K44}$ peptide against the neglected and drug-resistant pathogen *Candida krusei*

Thomas Z. A. Guimarães, Érica O. Mello, Douglas R. Lucas, Filipe Z. Damica, Fadi S. S. Magalhães, Luís G. M. Basso, André O. Carvalho, Valdirene M. Gomes, Gabriel B. Taveira*

Universidade Estadual do Norte Fluminense Darcy Ribeiro, Campos dos Goytacazes, RJ, postal code: 28013-602, Brazil

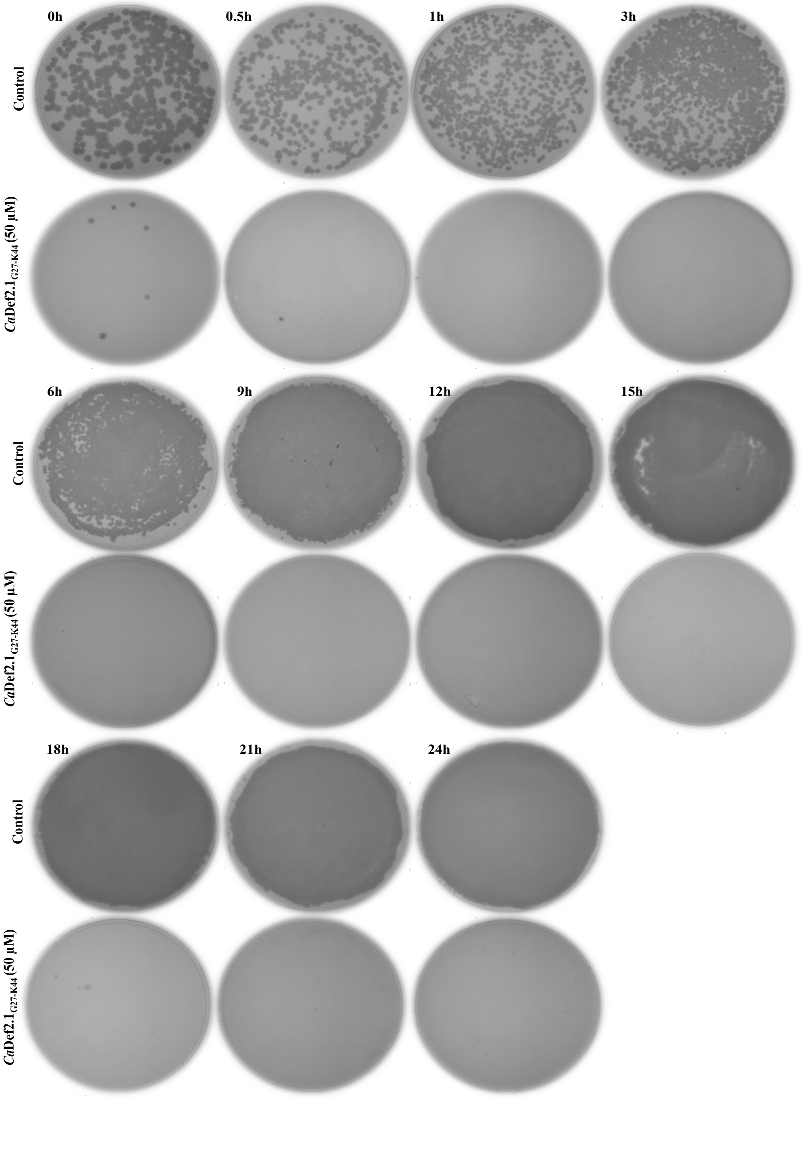

Figure S1. Kinetics of *C. krusei* yeast cell death. Petri dish images at each time point representing colony growth in the control (without CDF-GK) and after treatment with 50 µM CDF-GK. Experiments were performed in triplicate. Cell death kinetics were assessed over 24 h.

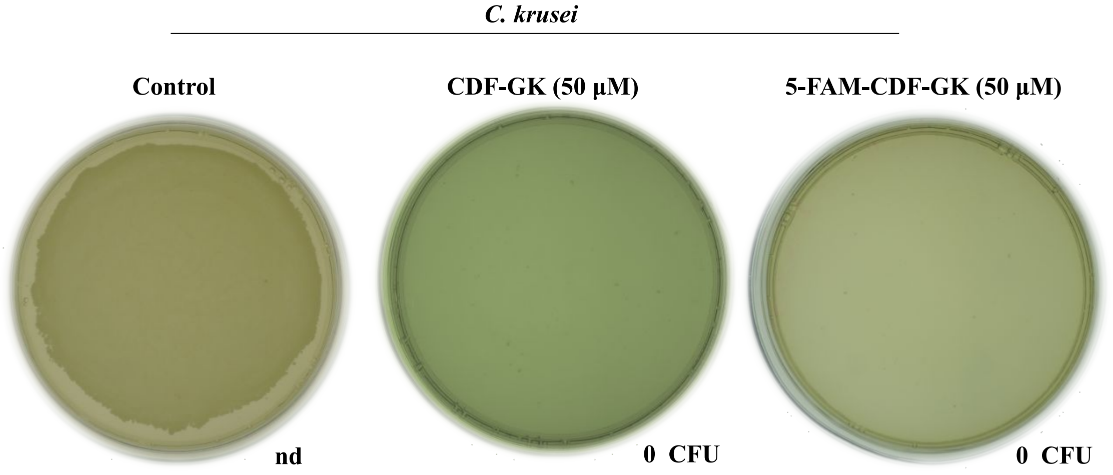

Figure S2. Fungicidal activity of CDF-GK coupled to 5-FAM at 50 µM against *C. krusei*. Control (untreated cells) showing robust colony formation. 5-FAM-CDF-GK at 50 µM (MFC_{100}), demonstrating complete eradication of colonies, equivalent to the activity of the unconjugated peptide CDF-GK at same concentration. Images are representative of two independent experiments. CFU: colony-forming units. nd: not determined.
